# Recent advances in the transition-metal-free synthesis of quinoxalines

**DOI:** 10.1039/d1ra06942j

**Published:** 2021-11-19

**Authors:** Biplob Borah, L. Raju Chowhan

**Affiliations:** School of Applied Material Sciences, Centre for Applied Chemistry, Central University of Gujarat Gandhinagar-382030 India rchowhan@cug.ac.in

## Abstract

Quinoxalines, also known as benzo[*a*]pyrazines, constitute an important class of nitrogen-containing heterocyclic compounds as a result of their widespread prevalence in natural products, biologically active synthetic drug candidates, and optoelectronic materials. Owing to their importance and chemists' ever-increasing imagination of new transformations of these products, tremendous efforts have been dedicated to finding more efficient approaches toward the synthesis of quinoxaline rings. The last decades have witnessed a marvellous outburst in modifying organic synthetic methods to create them sustainable for the betterment of our environment. The exploitation of transition-metal-free catalysis in organic synthesis leads to a new frontier to access biologically active heterocycles and provides an alternative method from the perspective of green and sustainable chemistry. Despite notable developments achieved in transition-metal catalyzed synthesis, the high cost involved in the preparation of the catalyst, toxicity, and difficulty in removing it from the final products constitute disadvantageous effects on the atom economy and eco-friendly nature of the transformation. In this review article, we have summarized the recent progress achieved in the synthesis of quinoxalines under transition-metal-free conditions and cover the reports from 2015 to date. This aspect is presented alongside the mechanistic rationalization and limitations of the reaction methodologies. The scopes of future developments are also highlighted.

## Abstract 2

Recent advances in the synthesis of quinoxalines under transition metal free conditions are summarized in this review article.

## Graphical abstract

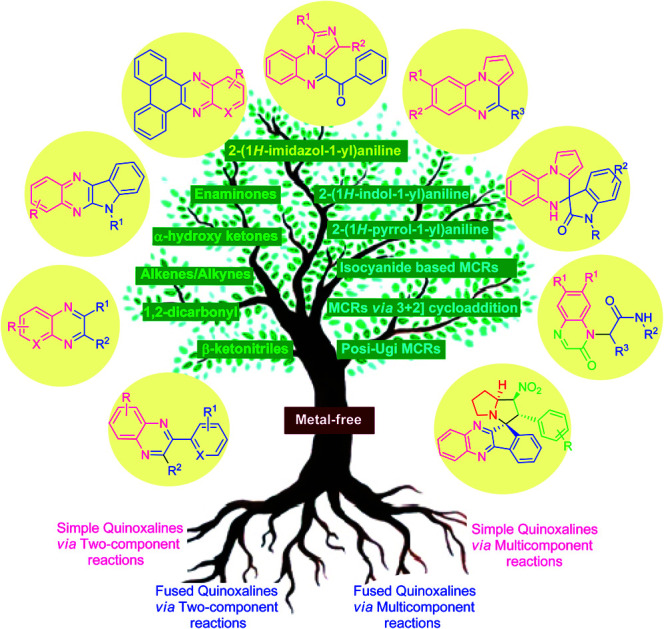

## 1. Introduction

Nitrogen-containing heterocycles, of natural and synthetic origin, represent a huge family of indispensable structural motifs and have attracted enormous attention in synthetic and medicinal chemistry as well as in material sciences owing to their wide-ranging chemical landscape and prolific bioactivity profiles.^1^ Among various nitrogen heterocycles, quinoxalines and their derivatives have been considered as versatile and privileged structural scaffolds in synthetic and medicinal chemistry.^2^ These well-established nitrogen-containing bicyclic heterocycles mainly consist of a benzene ring fused with a pyrazine ring in their structure. Quinoxalines structural motifs are widely distributed in the architecture of numerous natural products,^3^ pharmaceutical agents,^4^ agrochemicals,^5^ pharmacologically active synthetic compounds.^6^ They have been found to possess a diverse range of biological activities including antidiabetic,^7^ antimicrobial,^8^ anticancer,^9^ antituberculosis,^10^ antibacterial,^11^ anti-inflammatories,^12^ anti-HIV,^13^ antiviral,^14^ PI3Kγ inhibitors,^15^ cyclooxygenase 2 (COX-2) inhibitors,^16^ antimalarial,^17^ anti-dengue,^18^ antioxidant,^19^ anticonvulsant,^20^ antidepressant,^21^ antitumors,^22^ antiplasmodial,^23^ antileishmanial activity,^24^ and antiprotozoal activity.^25^

**Fig. 1:**
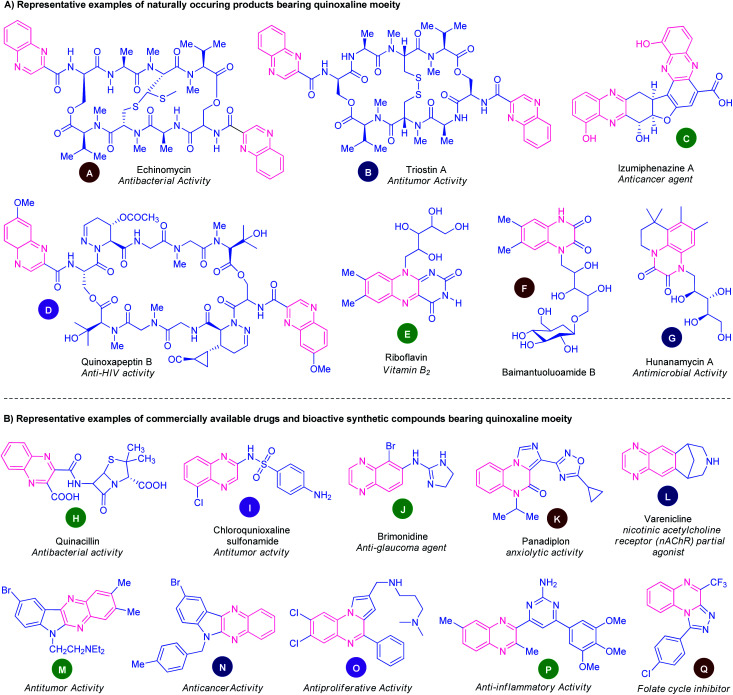
Representative examples of natural products, drugs, and bioactive synthetic compounds containing quinoxaline ring.

Several natural products that featured quinoxaline rings as an integral part are depicted in Fig. 1A. The natural product echinomycin (A) isolated from *Streptomyces echinatus sp.* was established for the first time in 1957.^26^ They have been found to possess anti-bacterial activity against Gram-negative organisms and have good anti-tumour activity. Another natural product triostin A (B) was isolated from *Streptomycesaureus* S-2-210 and has antitumor and antibacterial activity.^27^ Lumiphenazines (C) possesses anticancer activity was isolated from *Streptomyces sp*. IFM 11204.^28^ The natural product quinoxapeptin B (D) isolated from *Betula papyrifera* has been known for its anti-HIV activity.^29^ Quinoxalines are the building blocks of vitamin B_2_. Two marine alkaloids baimantuoluoamide B (F) and hunanamycin A (G) were isolated from alkaloidal fraction *Datura metel* L and *Bacillus hunanensis*.^30^

Besides these natural products, non-natural quinoxalines displayed prominent biological activities. In this pursuit, several quinoxaline-containing drugs and synthetic compounds of pharmacological profiles have been discovered (Fig. 1B). Quinacillin (H) is a quinoxaline-based semisynthetic penicillinase-resistant penicillin and has antibacterial activity.^31^ Chloroquinoxaline sulfonamide (I) is a topoisomerase II α/β-poison and is active against murine and human solid tumours.^32^ Brimonidine (J) is a commercially available α2 adrenergic agonist used to treat open-angle glaucoma, ocular hypertension, and rosacea.^33^ Panadiplon (K) acts as a high-affinity GABAA receptor partial agonist with anxiolytic activity.^34^ Varenicline (L) is a quinoxaline-based α4β2 nicotinic receptor partial agonist drug available in the market and is used for smoking cessation.^35^ Still, there are lots of quinoxaline derivatives possessing different biological activities which are under the stage of preclinical or clinical development. Several synthetic quinoxaline-based compounds M, N, O, P, and Q exhibited potent pharmacological activities such as antitumor,^36^ anticancer,^37^ antiproliferative,^38^ anti-inflammatory,^39^ and folate cycle inhibitors^40^ respectively.

Despite these wide-ranging biological activities, a diverse molecular structure that featured the quinoxaline framework has been established as potential fragments in diverse areas of materials science. The applications included organic photovoltaic devices,^41^ organic semiconductors,^42^ electroluminescent materials,^43^ fluorescent probes,^44^ organic light-emitting diodes (OLEDs),^45^ organic solar cells,^46^ sensitizers for dye-sensitized solar cells,^47^ polymer light-emitting diodes (PLEDs),^48^ and fuel cells.^49^

Considering the wide-ranging application and tremendous chemical landscape, a great deal of attention has been subjected toward the synthesis of quinoxalines in the last decades. However, most of the synthetic strategy utilizes hazardous reagents, toxic volatile solvents, harsh reaction condition, and high energy inputs that adversely affects the overall chemical process as well as the environment from being making eco-friendly and sustainable nature.

Despite, transition metal catalyst has been proven as a powerful catalyst and successfully employed in many organic synthesis for the diverse construction of valuable structural frameworks,^50^ they possess many limitations and drawbacks in terms of synthetic efficiency and green chemistry point of view. The high cost involved in the preparation of transition metal catalyst alongside the requirement of non-commercial supporting ligands marks major limitations of transition metal catalysis. In addition, they are very sensitive to air and moisture. The removal of transition metal catalysts from a reaction mixture which is particularly crucial to the pharmaceutical industry often becomes a formidable challenge and is very expensive. The requirements of co-catalysts and additives in some cases for enhancing the efficacy and reactivity of the transition metal-catalyzed transformation once again point towards the failure of green and more sustainable synthesis. Consequently, the occurrence of transition metal catalyst even at the lowest loading corresponds to disadvantageous effects on the eco-friendly and environmentally friendly nature of the chemical process.^51^ Intriguingly, the development of a chemical process that employed alternative materials for the synthetic purpose which are not only environmentally friendly but also readily accessible at bulk quantities in anywhere at a very low price, for the construction of structural complexity with high atom- and step-economy by avoiding the requirements of transition metal catalysts, co-catalyst, any additives are highly desired.

Recently, transition-metal-free reactions have been demonstrated as an efficient and environmentally benign strategy toward the synthesis of heterocyclic compounds and have been emerged as a key fascinating area in synthetic organic chemistry.^52^ They have several advantages in comparison to organic transformations involving transition metal catalysts. Consequently, tremendous growth has been witnessed in the last decade on the transition-metal-free synthesis of quinoxaline and its derivatives. However, to date, no review articles have summarized the development achieved in the synthesis of quinoxalines under transition-metal-free conditions.

Several review articles covered the synthetic strategy of quinoxalines starting from the classical one to non-conventional green ones and compiled them in the last few years.^53^ Recently, Maikhuri *et al.*, described the recent metal-catalyzed synthesis of quinoxalines.^54^ Also, Yashwantrao and Saha reviewed the synthesis and reactivity of quinoxaline derivatives.^55^ The present review article focuses on the recent progress achieved in the synthesis of quinoxalines and their fused structure under transition-metal-free conditions and covers the literature from 2015 to date. Besides, highlighting the so far advances realized in this fascinating area, we also point out the limitations and drawbacks of the strategy, and their possible scope of future developments has also been discussed. On behalf of a clear and understanding overview, the article is organized based on the different types of substrates used rather than compiled in chronological order.

## 2. Classical methods of quinoxaline synthesis

**Fig. 2:**
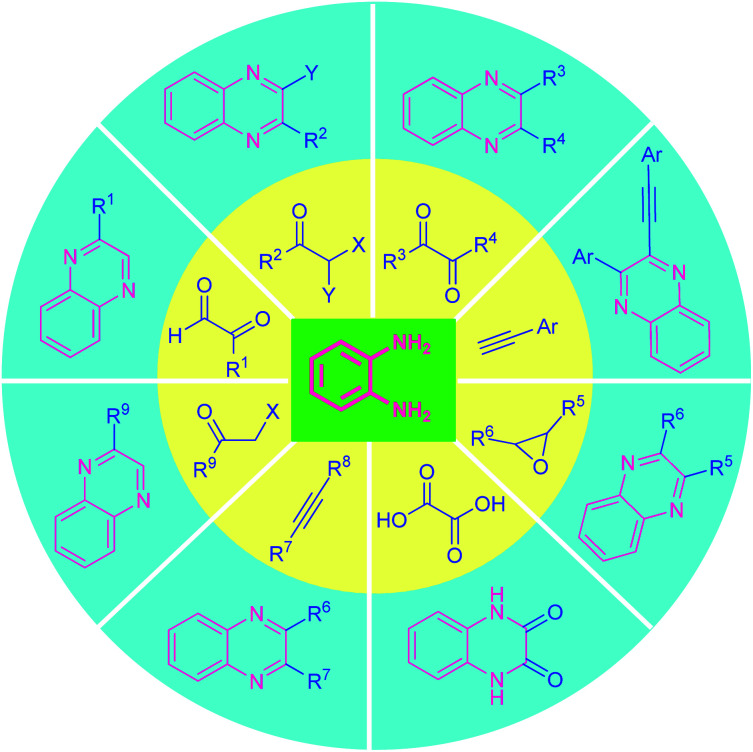
Various classical routes for the synthesis of quinoxalines derivatives.

Several synthetic strategies for the preparation of quinoxaline and its derivatives have existed since the late 1800's. The quinoxaline moiety is generally synthesized by employing classical methods including Körner^56^ and Hinsberg^57^ synthesis. Both of these reactions were independently reported in 1884, which mainly involves the condensation of *o*-phenylenediamine with synthons possessing reactive 1,2-dicarbonyl core (*e.g.*, 1,2-diketones, 1,2-ketoesters, or oxalic acid derivatives).^58^ A constructive modification of these synthetic methods introduces 1,2-dicarbonyl compound surrogates (*e.g.*, α-haloketones, α-hydroxyketones, epoxides, or alkynes)^58^ (Fig. 2).

Though many of these methods are very attractive in terms of product yield as well as broad functionality, they often utilize various acids, metals, co-catalyst, or reagents that are not environmentally friendly, and produces a large amount of waste as well as hazardous by-products which is difficult to dispose of properly and remove from the reaction mixture and leads to the contamination of the products and the reaction required unusually longer reaction times to complete. Consequently, tremendous growths have been witnessed in the last few years for the metal-free synthesis of quinoxalines, and are compiled in this review.

## 3. Synthesis of simple quinoxalines based on metal-free two-component reactions

Synthesis of quinoxalines based on a metal-free approach can be accomplished *via* different reaction types and pathways. Most of them proceed through the modification of classical strategy and newly developed strategy.

### 3.1 From 1,2-dicarbonyl compounds

**Scheme 1:**
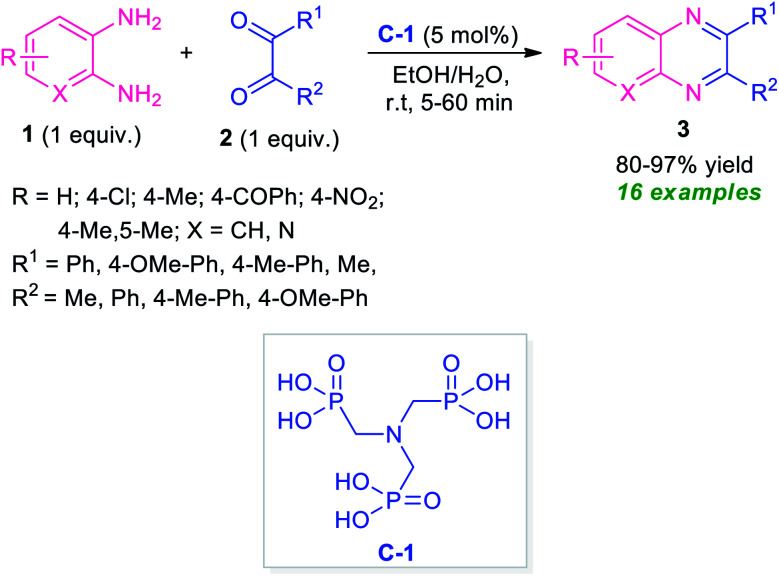
Efficient organocatalytic approach towards the rapid access of quinoxaline derivatives 3.

After the renaissance of organocatalyst, the field of organic synthesis reached an excellent level from the perspective of green and sustainable chemistry. Inspired by the development achieved in the organocatalysis, Fathi and Sardarian demonstrated an efficient organocatalytic approach for the synthesis of various quinoxalines 3 from the reaction of 1,2-diamines 1 and 1,2-carbonyl compounds 2 (Scheme 1).^59^ With the help of 5 mol% of nitrilotris(methylenephosphonic acid) C-1 as the organocatalyst, the corresponding quinoxaline products have been synthesized in 80–97% yields within a very short reaction time. The reaction condition tolerates a wide variety of substituents on the 1,2-diamine ring as well as on the 1,2-dicarbonyl ring. The efficacy of the protocol was established by demonstrating the recyclability and reusability of the organocatalyst up to several consecutive cycles with a slight change in the product yield.

**Scheme 2:**
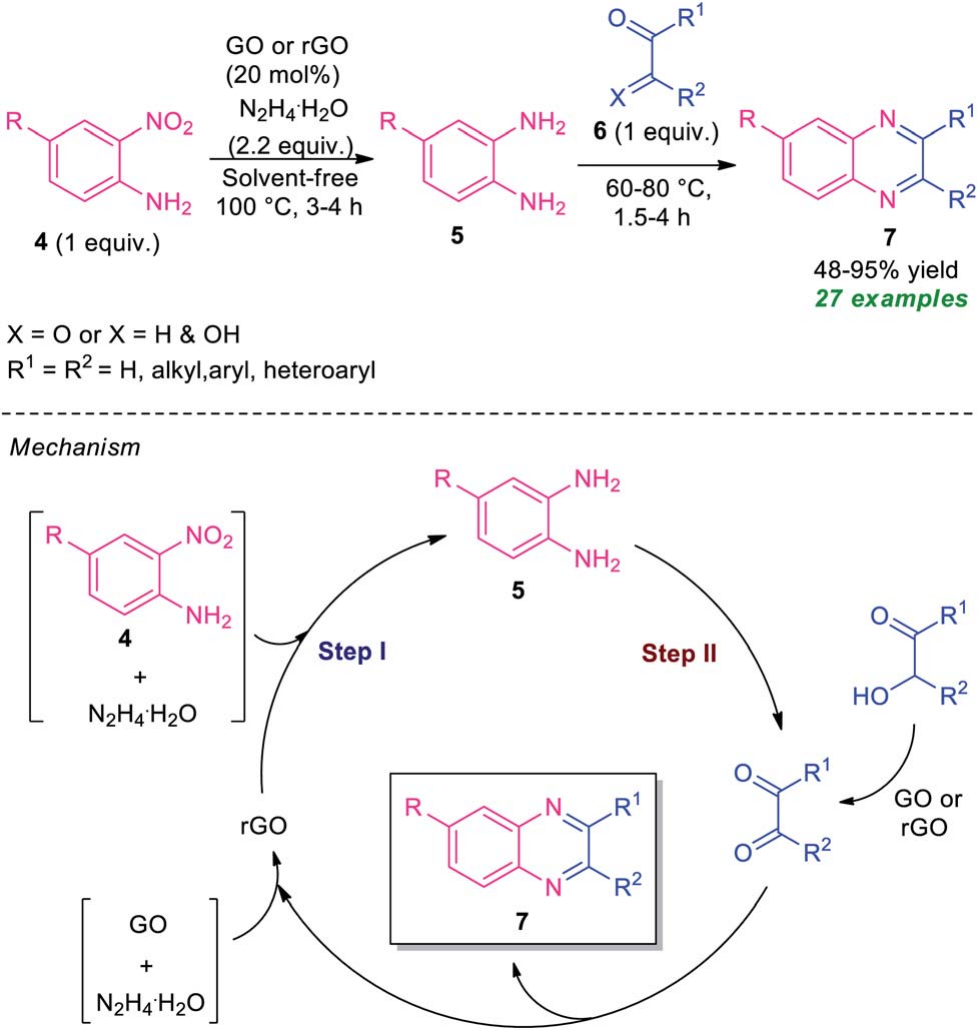
One-pot reduction and subsequent tandem condensation to access diverse quinoxaline derivatives 7.

At the same time, Basu and co-workers disclosed a metal-free one-pot synthesis of quinoxalines 7 from 2-nitroaniline by using graphene oxide (GO) or reduced graphene oxide (rGO) as the carbon catalyst (Scheme 2).^60^ The reaction proceeded through the initial graphene oxide catalyzed reduction of 2-nitroaniline 4 with hydrazine hydrate and subsequent one-pot tandem reaction with 1,2-dicarbonyl compounds or α-hydroxy ketones 6. Twenty-seven derivatives were synthesized in moderate to excellent yield. The catalyst was easily recovered and reused up to four runs without affecting the product yield.

A mechanism was proposed by the author to explain this transformation which was depicted in Scheme 2. The first step of the reaction involves the reduction of 2-nitroaniline 4*via* a four-electron pathway which utilized two-electron reducing agent hydrazine to afford the 1,2-diamine 5 where GO acts as an adsorbent as well as a collector of hydrazines in its surface. In the second step, the catalyst GO or rGO effectively facilitates the condensation of 1,2-dicarbonyls as well as oxidation of α-hydroxyketones to corresponding 1,2-dicarbonyls for subsequent condensation to final products 7.

**Scheme 3:**
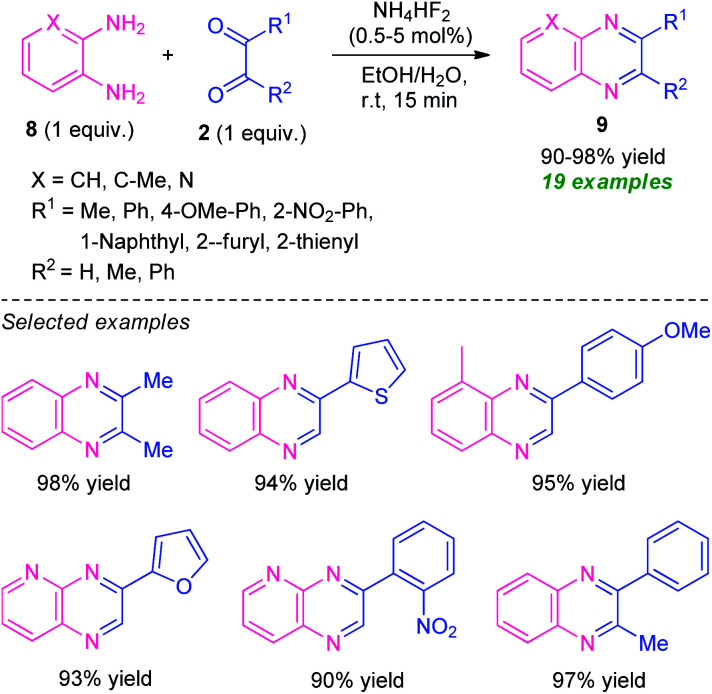
Ammonium bifluoride catalyzed synthesis of various quinoxalines 9.

Treatment of various *o*-phenylenediamine 8 and 1,2-dicarbonyl compounds 2 was found to proceed under the influence of ammonium bifluoride (NH_4_HF_2_) as the catalyst and aqueous ethanol as the solvent system to efficiently provide the corresponding quinoxaline derivatives 9 in 90–98% yields (Scheme 3).^61^ A variety of alkyl, aryl- and heteroaryl-substituted 1,2-dicarbonyl compounds as well as substituted *o*-phenylenediamine participate in the reaction smoothly under this mild reaction condition. With unsymmetrical dicarbonyl compounds and *o*-phenylenediamine, the products were formed regioselectively in excellent yield. The mild reaction condition, simple work-up procedure, low catalyst loading, utilization of easily available starting material, recyclability for further reaction cycles with negligible loss in catalytic property, are some of the key features of this approach.

**Scheme 4:**
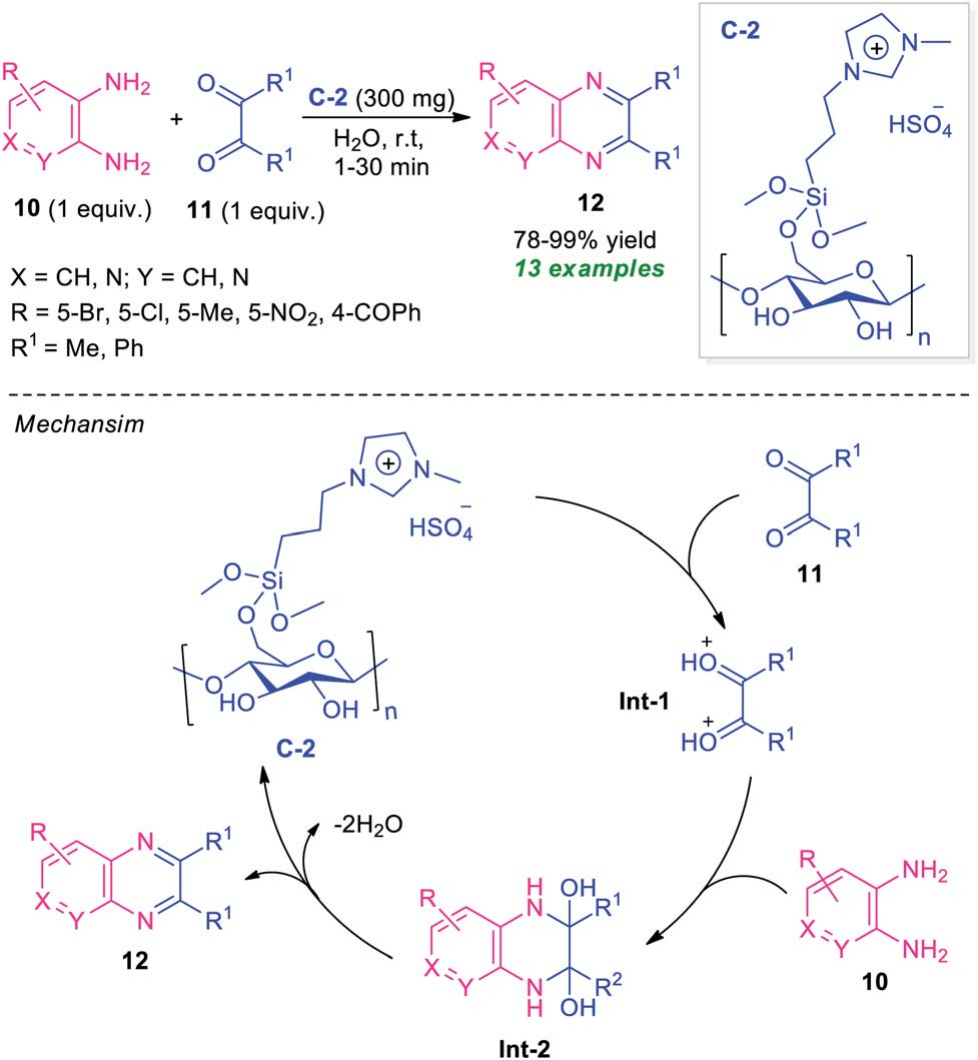
Ionic liquid functionalized cellulose as an efficient catalyst for the rapid access to quinoxalines 12.

Recently, the utilization of ionic liquid as a catalyst as well as a solvent system in organic synthesis has attracted much more attention as they provide green alternatives to metal catalysts and hazardous organic solvents.^62^ In this regard, Moghaddam and Valizadeh designed and prepared ionic liquid 1-methyl-3-(3-trimethoxysilylpropyl) imidazolium hydrogen sulfate functionalized cellulose C-2 as a heterogeneous catalyst, and the catalytic activity was examined in the reaction of diverse *o*-phenylenediamine 10 with 1,2-dicarbonyl compound 11 (Scheme 4).^63^ The prepared catalyst C-2 was found to be very effective in catalyzing this condensation reaction to afford the desired quinoxaline products 12 in 78–99% yields. This reaction required only 300 mg of the catalyst and by using water as the green solvent, a total of 13 compounds were synthesized.

The reaction can proceed through the catalytic cycle as depicted in Scheme 4. Initially, the Brønsted acid catalyst C-2 enhances the nucleophilic character of 1,2-carbonyl compounds thereby facilitating the protonation of the carbonyl groups. The resulting intermediate Int-1 experiences a nucleophilic attack from the nitrogen of diamines to deliver the intermediate Int-2, which is followed by dehydration to yield the final product 12.

Considering the importance of quinoxaline rings along with the advantages associated with the transition-metal-free catalysis, several eco-friendly and environmentally benign strategies for the synthesis of quinoxaline derivatives 3 from *o*-phenylenediamine 1 and 1,2-dicarbonyl compounds 2 has been developed. All the reactions were performed in the presence of different catalytic systems including ionic liquid, bio-based organocatalyst, green catalyst as well as under catalyst-free conditions. The utilization of ethanol, aqueous ethanol, or solvent-free conditions makes the developed methodology green and sustainable. The products were obtained in moderate to excellent yield within a very short reaction time in almost all of the cases. All the reactions required a low amount of catalyst which marks the salient features of the developed methodologies. Broad functional group tolerance, mild reaction condition, recoverability, and reusability of the catalyst without affecting the outcome of the reaction, are some of the advantages of all of the developed strategies (Table 1).^64–70^

**Table 1:** Reaction of various *o*-phenylenediamine 1 with 1,2-dicarbonyl compounds 2 under different reaction conditions for the synthesis of diverse quinoxaline derivatives 3

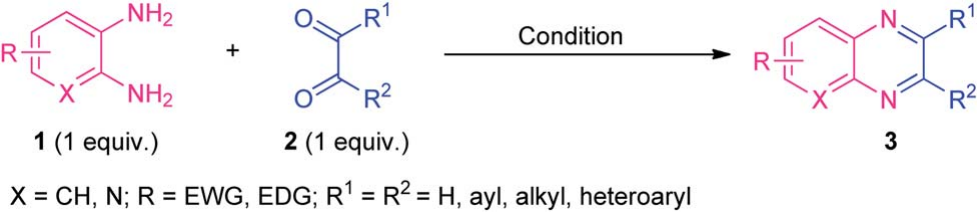								
Entry	Catalyst	Loading	Solvent	Condition	Time (min)	Examples	Yield (%)	Ref.
1	DABCO : AcOH : H_2_O^a^	5 mol%	Solvent-free	80 °C	20–45	12	86–98	64
2	PSA^b^	5 mol%	EtOH	r.t	10–30	15	88–95	65
3	RHA-SO_3_H^c^	15 mg	Solvent-free	r.t	5–30	11	90–98	66
4	Vitamin C	11 mol%	EtOH	r.t	2–100	17	60–98	67
5	Sulfated polyborate	10 wt%	Solvent-free	100 °C	3–10	18	95–99	68
6	Catalyst-free	—	EtOH	Grinding, r.t	10–30	30	80–98	69
7	GA^d^	0.05 g	EtOH : H_2_O (4 : 1)	r.t	0.6–18^e^	11	55–98	70

a 1 : 1 : 3 ratio is used.

b Phospho sulfonic acid.

c sulfonated rice husk ash.

d Gum arabic.

e Time expressed in hours.

Although their emergence in organic chemistry in only about the last decades, ultrasound irradiation has been considered as a powerful alternative energy source for the construction of value-added compounds to make them sustainable from the perspective of green chemistry.^71^

**Scheme 5:**
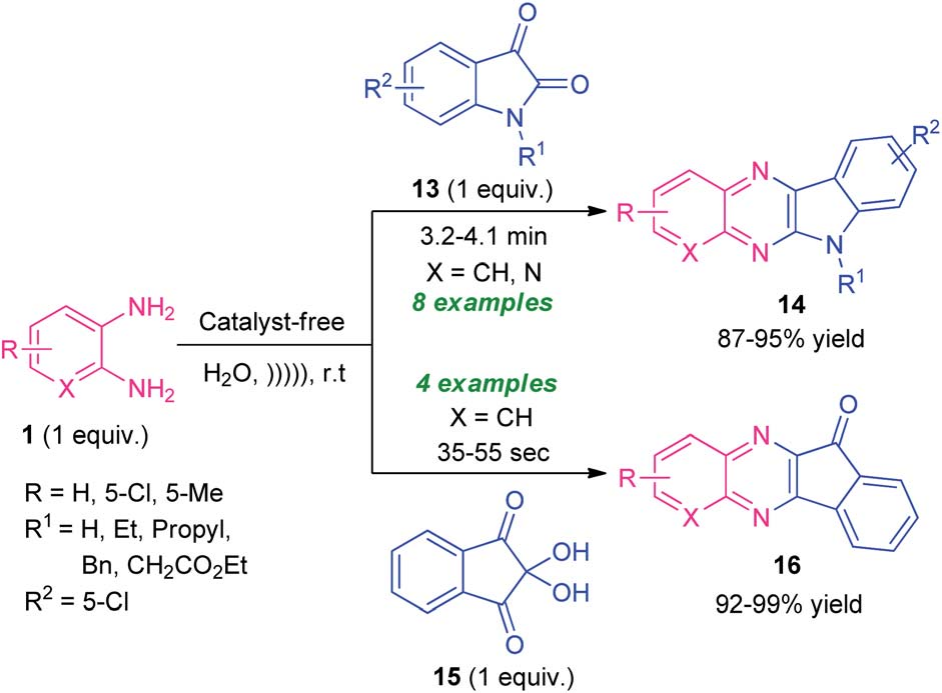
Ultrasound irradiated catalyst-free synthesis of diverse quinoxaline derivatives 14 and 16.

Given the importance of ultrasound irradiation in organic synthesis, Srivastava and co-workers in 2019, demonstrated an ultrasound irradiated catalyst-free protocol for the synthesis of diverse fused quinoxaline derivatives from several substituted *o*-phenylenediamines by utilizing water as the reaction medium (Scheme 5).^72^ Treatment of *o*-phenylenediamine 1 with isatin 13 under this optimized condition found to proceed smoothly to afford indolo[2,3-*b*]quinoxalines 14 in 87–95% yields; whereas the reaction of diamine 1 with ninhydrin 15 delivered the indeno[1,2-*b*]quinoxaline derivatives 16 in 92–99% yields within very short reaction time. The scope of the reaction was established by varying different substitutions on the diamine ring and isatin ring. It is interesting to note that, unsubstituted isatin (R^1^ = H) as well as substituted isatin (R^1^ = Et, Bn, Pr) were well tolerated by this approach.

**Scheme 6:**
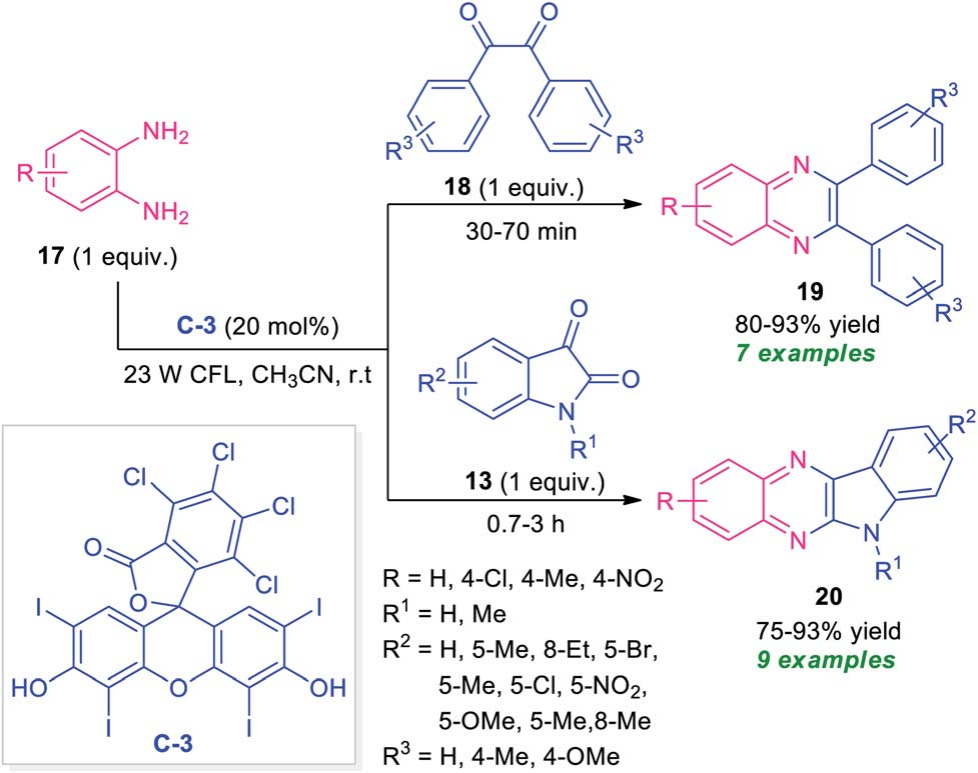
Visible light-mediated Rose Bengal catalyzed synthesis of different quinoxaline derivatives 19 and 20.

**Scheme 7:**
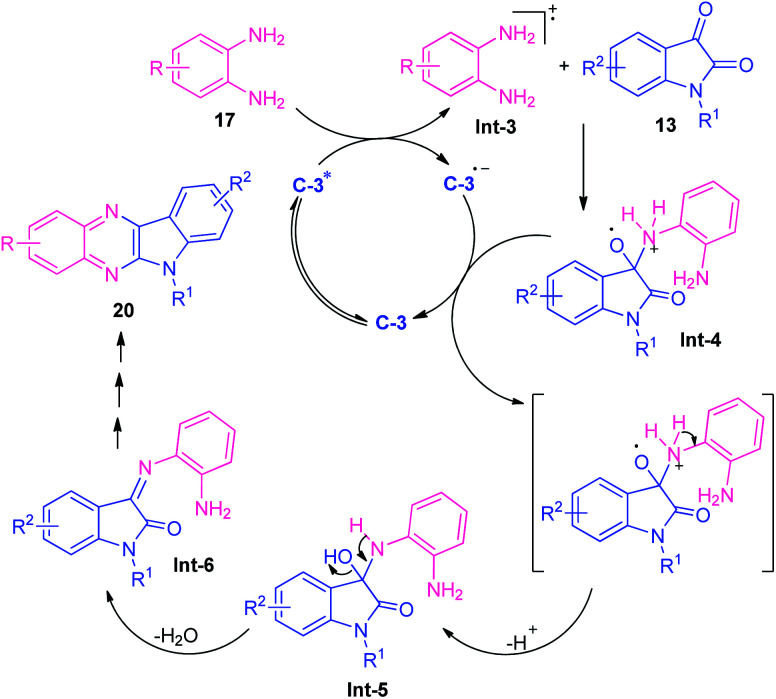
The suggested mechanism to explain the formation of quinoxalines 20.

At the same time, Singh and their groups disclosed a visible light promoted organo-photoredox catalyzed chemoselective strategy toward the synthesis of a variety of quinoxalines (Scheme 6).^73^ With the help of 20 mol% of Rose Bengal C-3 as the organo-photoredox catalyst, the treatment of 1,2-diamines and 1,2-dicarbonyl compounds including substituted benzil 18 or substituted isatin 13 were found to take place at room temperature to afford the desired quinoxaline products 19 and 20 in moderate to excellent yield respectively. The mechanism to explain this reaction has been covered in Scheme 7. Initially, the photoredox catalyst C-3 is excited to its singlet state under visible light irradiation, which can be further excited to the triplet state through intersystem crossing (ISC). Pleasingly, under influence of this triplet state, 1,2-diamine converts into radical-cation Int-3 which reacts with isatin to form intermediate Int-4. Subsequent single electron transfer (SET) from C-3˙^−^ followed by removal of proton results in the formation of intermediate Int-5 from Int-4, which on dehydration afforded the intermediate Int-6. The cyclization of Int-6 leads to the desired products 20.

**Scheme 8:**
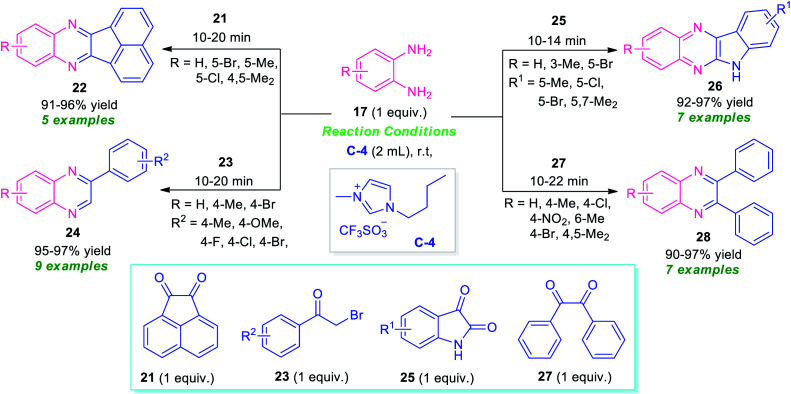
Preparation of library of quinoxaline derivatives in ionic liquid.

Bhargava *et al.*, synthesized a series of diverse quinoxaline derivatives 22, 24, 26, and 28 from the equimolar amount of *o*-phenylenediamine 17 and different types of carbonyl substrates such as acenaphthoquinone 21, substituted bromoacetophenone 23, isatin 25, and benzil 27 by using ionic liquid C-4 as the catalyst as well as reaction medium (Scheme 8).^74^ Interestingly, the products were obtained in good to excellent yield within a very short reaction time at room temperature in all the cases. The effectiveness of the protocol was established by demonstrating the reactivity of recovered ionic liquids for up to six reaction cycles without loss in its catalytic activity. Also, wide substrates scope, high yields, simple work-up procedure, and short reaction time make this approach very efficient and environmentally benign.

**Scheme 9:**
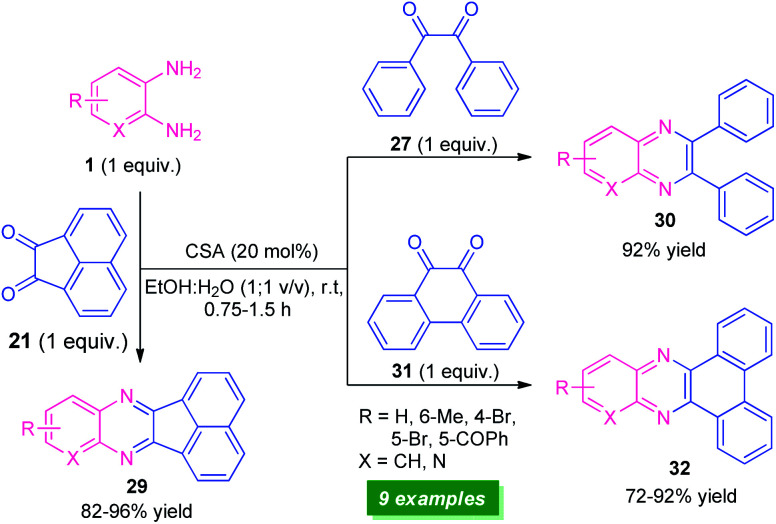
Camphor sulfonic acid-catalyzed synthesis of quinoxalines 29, 30, and 32.

Recently, a straightforward organocatalytic strategy for the synthesis of different types of quinoxaline derivatives was developed by Banerjee's group (Scheme 9).^75^ By using 20 mol% of camphor sulfonic acid as the organocatalyst, various quinoxaline derivatives 29, 30, and 32 derived from easily available starting material *o*-phenylenediamine 1 and carbonyl substrates acenaphthylene-1,2-dione 21, benzil 27, and phenanthrene-9,10-dione 31, have been obtained in moderate to excellent yield within a very short reaction time. Utilization of commercially available, camphor sulfonic acid as an organocatalyst, aqueous ethanol as the solvent, mild reaction conditions make this protocol environmentally and eco-friendly benign. However, low substrate scopes mark a major limitation of this approach and demand further developments otherwise outstanding work.

### 3.2 From α-halo-ketones

Owing to the dual functionality as they contain two electrophilic sites, α-halo-ketones are recently gained as important building blocks for the synthesis of numerous active heterocycles as well as undergo various types of reaction.^76^ α-Halo-ketones mainly phenacyl bromides were utilized in the synthesis of quinoxaline derivatives.

**Scheme 10:**
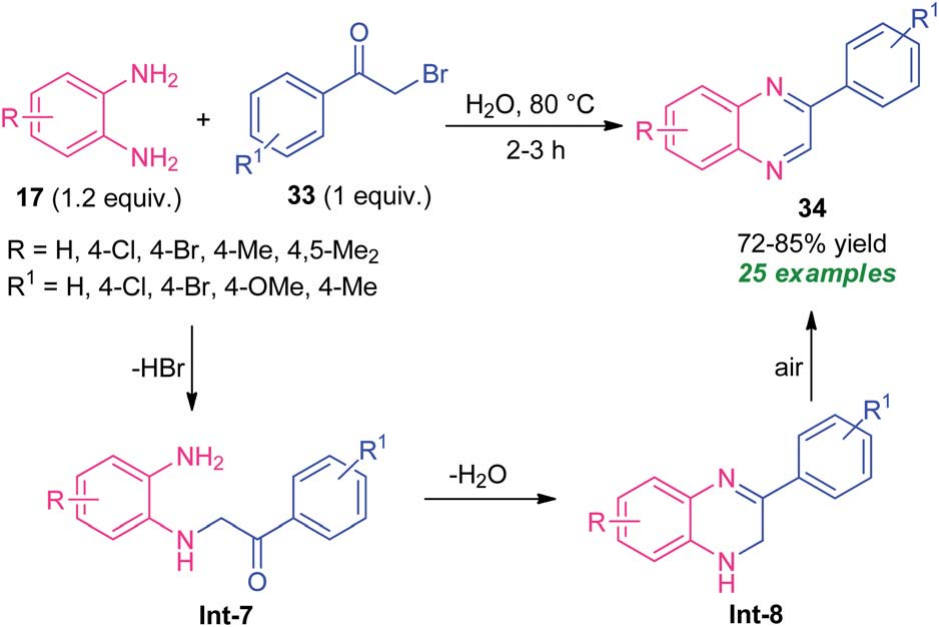
Catalyst-free oxidative cyclization of diamines and phenacyl bromide to access quinoxalines.

In 2015, a highly efficient and practicable one-pot strategy toward the synthesis of quinoxalines *via* oxidative cyclization of α-halo ketones and *o*-phenylenediamine was developed by Nair and their groups (Scheme 10).^77^ Treatment of several diamines 17 with substituted phenacyl bromide 33 in water at 80 °C afforded the desired quinoxalines 34 in moderate to high yield. This reaction does not require any catalyst as well as co-catalyst, and additives. Phenacyl bromide-bearing electron-donating, as well as electron-withdrawing substituents and symmetrical diamines, smoothly underwent the reaction under this condition. However, when unsymmetrical diamines were employed, the products were formed regioselectively. The overall process can initiate through the nucleophilic attack of diamines 17 with phenacyl bromide 33 to produce the intermediate Int-7 that can then be cyclized into intermediate Int-8. Subsequently, the aromatization of intermediate Int-8 was occurred in air oxidation to afford the final products 34.

**Scheme 11:**
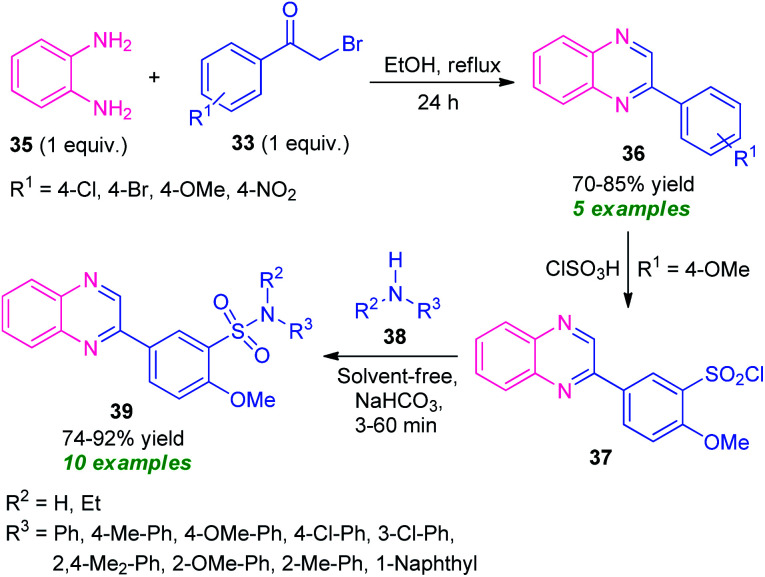
Three-step synthesis of quinoxaline-sulfonamides based on a green catalyst-free strategy.

Massah *et al.*, in 2017, disclosed a catalyst-free protocol for the synthesis of quinoxaline derivatives 38 from the reaction of *o*-phenylenediamine 35 and phenacyl bromide 33 under reflux condition using ethanol as the green solvent (Scheme 11).^78^ The products were obtained in 70–85% yields. The synthesized quinoxaline derivatives were extended to quinoxaline sulfonamides 39*via* a two-step reaction. The first step involves the reaction of quinoxaline 36 (R^1^ = OMe) with ClSO_3_H to afford the quinoxaline sulfonyl chloride 37. Treatment of 37 with different aryl amines 38 under solvent-free conditions lead to the final quinoxaline sulfonamides 39*via* the second steps. The respective products were achieved in moderate to high yield. Most of the synthesized compounds have been established as good antibacterial agents against *Staphylococcus* spp. and *Escherichia coli bacteria.*

### 3.3 From β-ketonitriles

Similar to α-halo-ketones, β-ketonitriles represent an important class of highly reactive precursors for the preparation of diverse biologically active pharmaceuticals and heterocyclic compounds due to the wide functionality of ketone and nitrile groups present on it.^79^

**Scheme 12:**
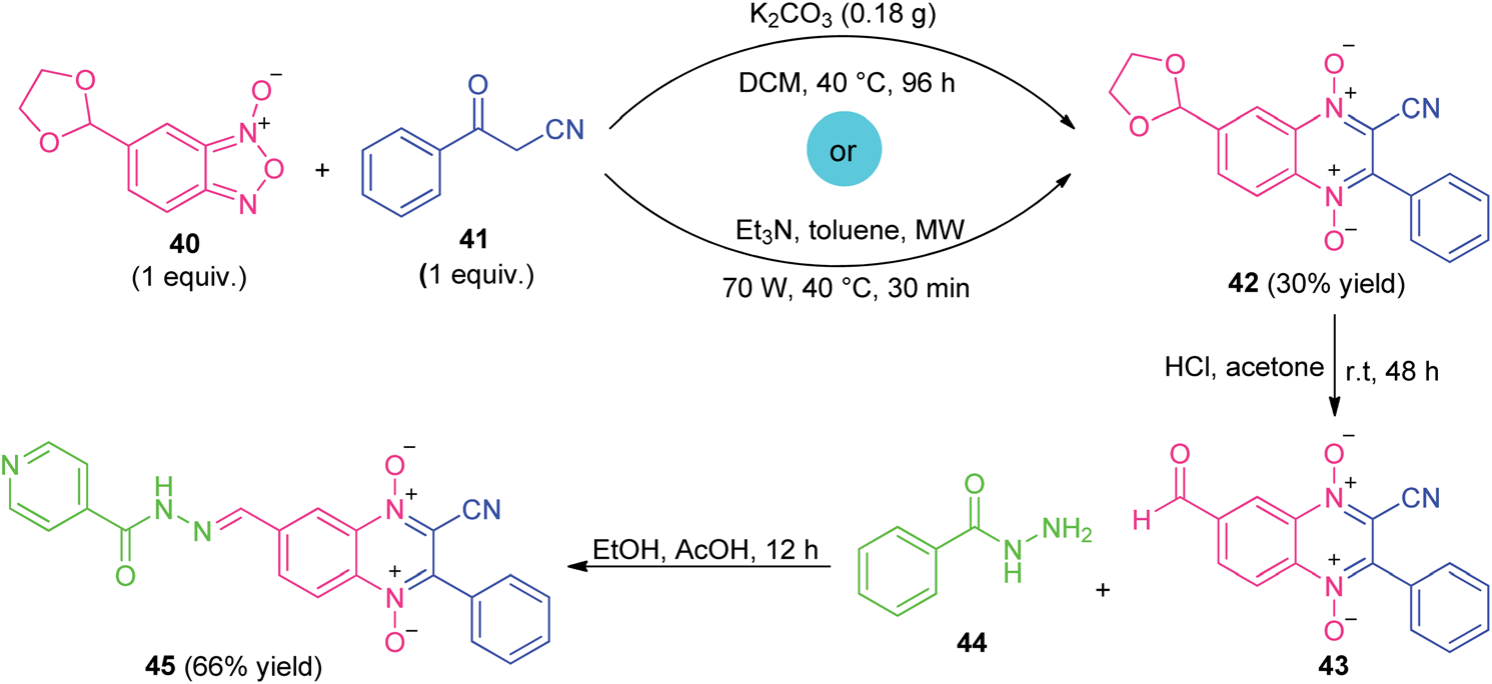
Metal-free conventional as well as the microwave-assisted synthesis of quinoxaline hybrid 45.

Considering their versatility, along with the prevalence of quinoxaline-1,4-di-*N*-oxide (QdNO) and *N*-acylhydrazone in medicinal chemistry, Santos *et al.*, realized a metal-free step-wise approach for the synthesis of quinoxaline hybrid 45 through the Beirut reaction sequence (Scheme 12).^80^ Initially, the reaction of dioxolan-benzofuroxan 40 with benzoylacetonitrile 41 was successfully carried out *via* conventional as well as microwave-irradiation to afford the adduct 42 under the influence of either K_2_CO_3_ or Et_3_N as metal-free bases. Although, both the method provides the same amount of yield of adduct 42 (30%), however, microwave technique was found to be very advantageous over conventional one in terms of reaction time. The subsequent deprotection of 42 by acid hydrolysis and *in situ* condensation of resulting adduct 43 with isoniazid 44 lead to the final quinoxaline-1,4-di-*N*-oxide-*N*-acylhydrazone 45 in 66% yields.

**Scheme 13:**
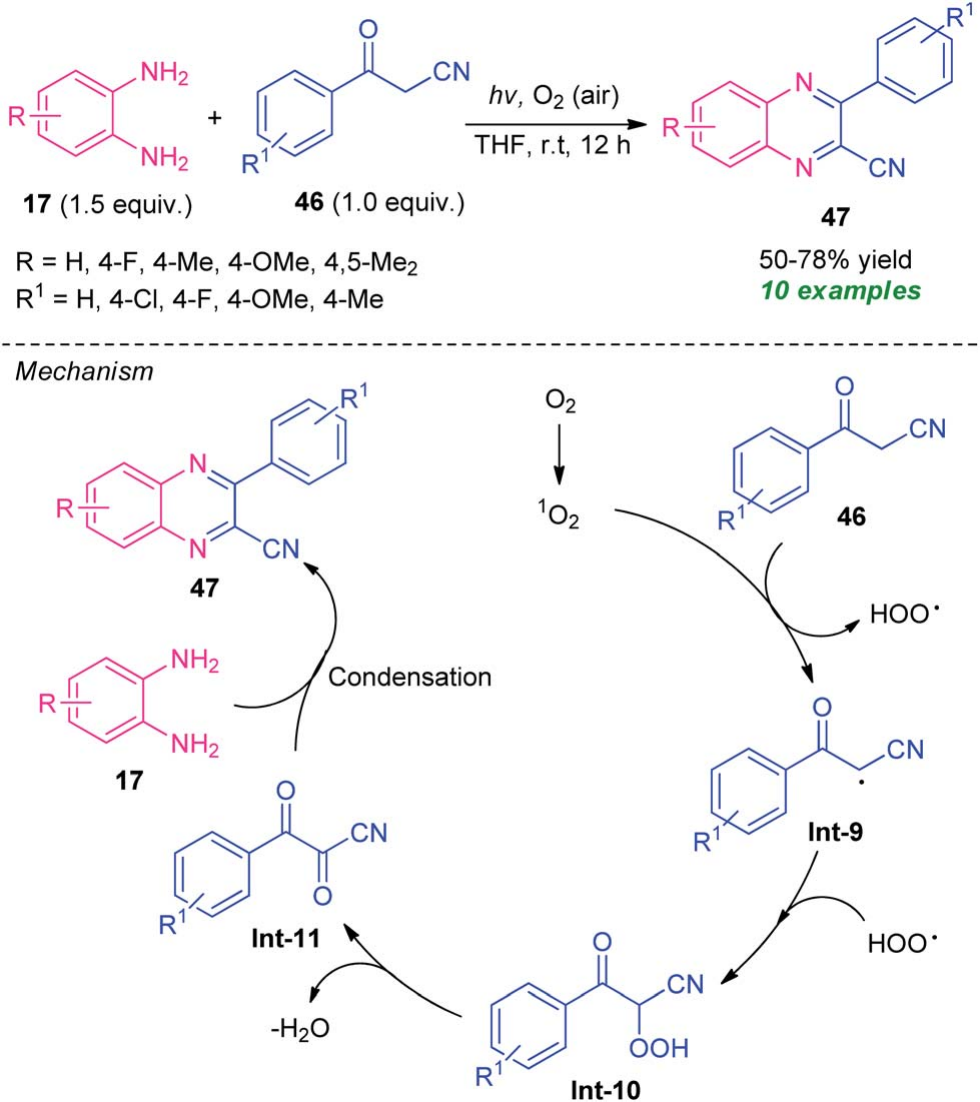
Visible light-induced electron-transfer and oxidative cyclization to access quinoxaline derivatives.

A highly efficient visible-light mediated metal-free synthesis of quinoxalines from easily available *o*-phenylenediamine 17 and benzoylacetonitrile 46*via* single electron-transfer and oxidative coupling strategy has been developed by Guo *et al.*, in 2019 (Scheme 13).^81^ Pleasingly, a total of 10 compounds were synthesized in 50–78% yields by this mild reaction condition at room temperature. A variety of substitutions on the diamine ring as well as on the benzoylacetonitrile were found to effectively work by this approach. The mechanistic pathway behind this transformation involves the reaction of benzoylacetonitrile 46 with singlet oxygen generated from the oxygen under the influence of visible light, to produce a radical intermediate Int-9. The intermediate Int-9 then reacted with HOO radical and deliver the intermediate Int-10 which underwent dehydration to afford the intermediate Int-11.

Consequently, condensation of Int-10 with diamine 17 leads to the final product 47.

### 3.4 From α-hydroxy ketones/diols

Given the importance of α-hydroxy ketones in the compounds of the pharmaceutical interest and optically active value-added compounds, the synthesis and their derivatization into potential lead compounds has received tremendous attention in organic synthesis.^82^

**Scheme 14:**
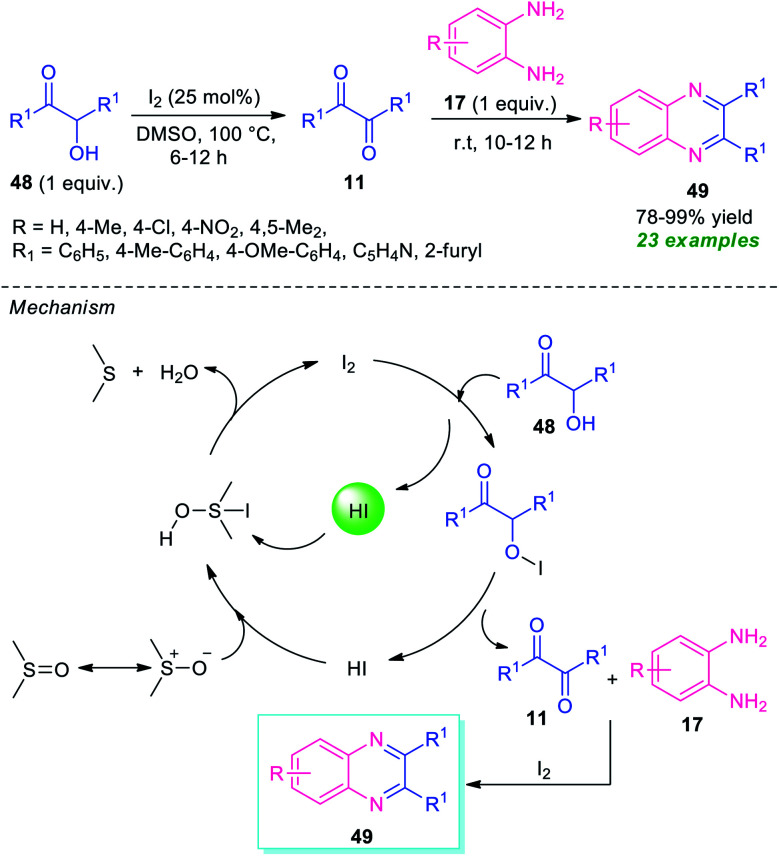
Iodine catalyzed one-pot oxidation/cyclization to access quinoxalines 49.

In 2015, Ma and his group demonstrated a straightforward one-pot two-step procedure for the synthesis of quinoxaline derivatives 49 from α-hydroxy ketones 48 (Scheme 14).^83^ With the help of 20 mol% of I_2_ as the catalyst and DMSO as the solvent as well as oxidant, the corresponding quinoxaline products 49 have been synthesized in 78–99% yields. Different substituted *o*-phenylenediamine 17 was found to proceed smoothly under this condition. A total of 23 compounds were synthesized by this method. Broad functional group tolerances, excellent level of yield, metal-free reaction conditions are some of the salient features of this approach. This process starts with the initial oxidation of hydroxy ketones 48 to the desired dicarbonyl compound 11 under the influences of I_2_ as the oxidant. Then the subsequent condensation of 11 with diamine 17 takes place smoothly, to form the final product 49 under the influence of I_2_ that acts as Lewis acid in this step. For recycling the catalytic system, HI was oxidized into iodine with DMSO as the oxidant, and then iodine was regenerated for further operation.

**Scheme 15:**
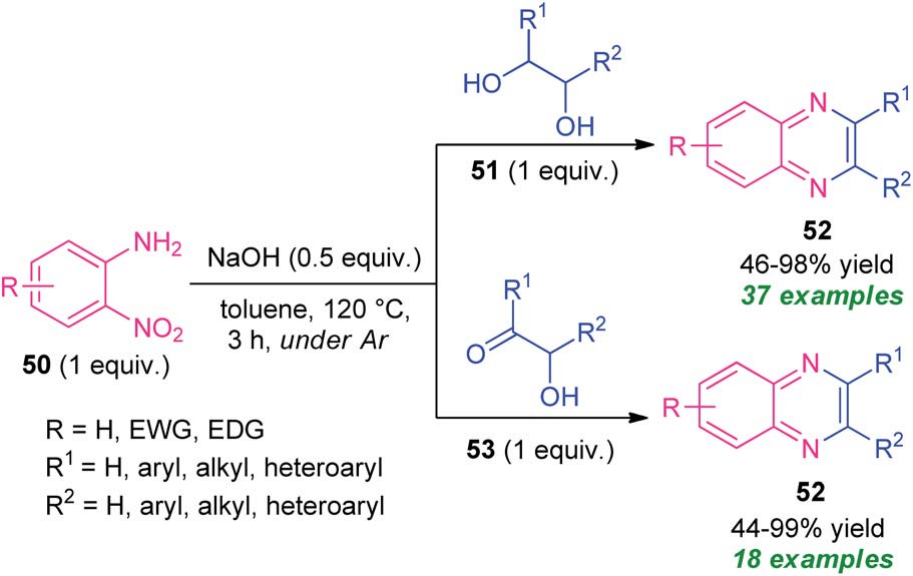
Transition-metal-free redox condensation reaction to access quinoxaline derivatives 52.

In 2020, a transition-metal-free highly convenient strategy for the construction of quinoxaline derivatives *via* redox condensation of 2-nitroamines with α-hydroxy ketone or diols was disclosed by Zhu, Song, and their groups (Scheme 15).^84^ For optimizing the reaction condition, the reaction was initially carried out under the influence of different bases including NaOH, NaO^*t*^Bu, KO^*t*^Bu, KOH as well as in different solvent systems like toluene, *o*-xylene, dioxane, and also in solvent-free conditions. Among them, the exploitation of NaOH as the base catalyst in toluene was selected as the standard condition. Under this condition, the treatment of 2-nitroamine 50 with diols 51 afforded various quinoxaline derivatives 52 in moderate to excellent yields (46–98%), whereas the reaction of 2-nirtoamine 50 and α-hydroxy ketone 53 delivered the corresponding product 52 in 44–99% yields. By employing both the substrates a total of 55 compounds were has been synthesized.

**Scheme 16:**
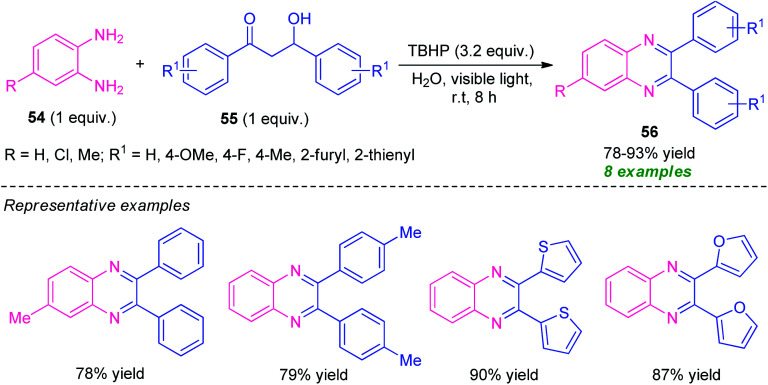
Metal-free visible light-mediated domino synthesis of quinoxalines 56.

Later, Sarma and co-workers disclosed a visible light-assisted catalyst-free green protocol toward the synthesis of a series of quinoxaline derivatives 56 by employing easily accessible *o*-phenylenediamine 54 and α-hydroxy ketone 55 as the starting material (Scheme 16).^85^ With the help of *tert*-Butyl hydroperoxide (TBHP) as the oxidizing agent, the corresponding quinoxaline products 56 were obtained in 78–93% yields. The decomposition of TBHP into its radical was predominately promoted by metal or photocatalysts, or other reagents, however in this reaction, the decomposition of TBHP for the radical formation and its acceleration for the completion of the reaction was achieved simply by visible light irradiation and no extra metal- or photocatalyst is required. The successful utilization of photoinduced catalyst-free protocol in an aqueous medium makes this approach very efficient toward green and sustainable practices.

### 3.5 From alkenes

**Scheme 17:**
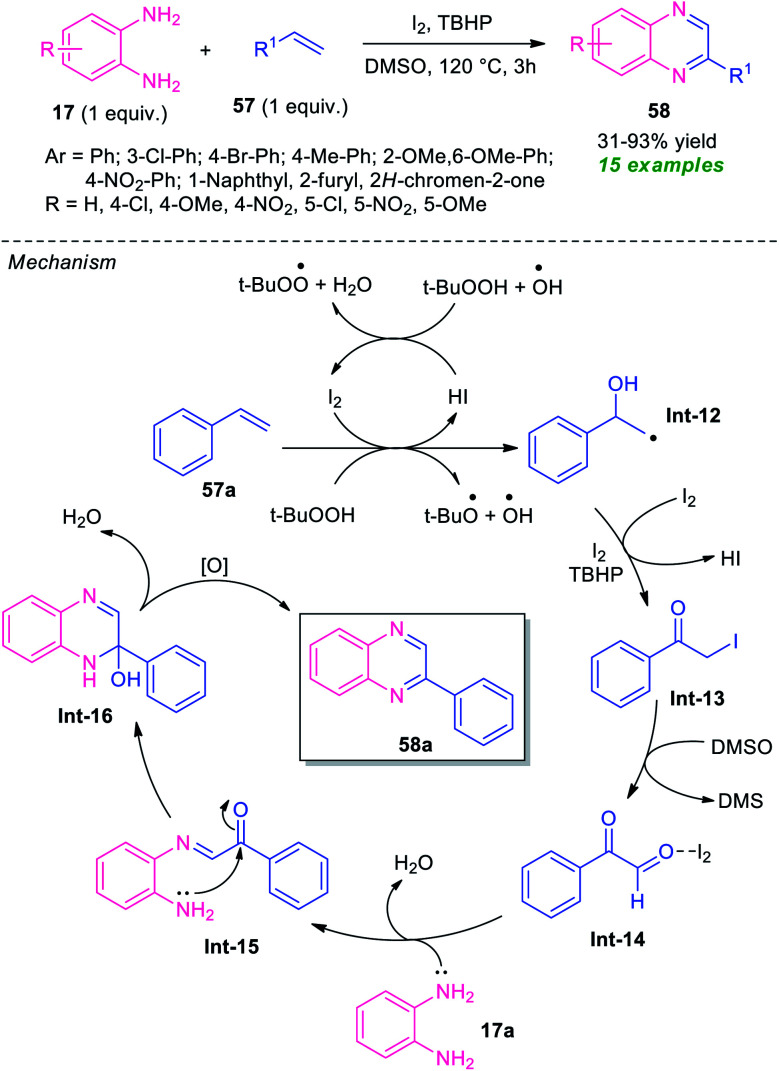
I_2_-catalyzed domino one-pot atom-economic approach to quinoxaline derivatives 58.

The utilization of alkenes in the metal-free domino synthesis of quinoxaline derivatives has been achieved by Chaskar *et al.*, in 2015 (Scheme 17).^86^ Using I_2_ as the catalyst and TBHP as the oxidant in DMSO, the corresponding quinoxaline products 57 derived from various *o*-phenylenediamine 17 and alkenes 57, were obtained in 31–93% yields. Not only aromatic alkenes but also heteroaromatic alkenes were well tolerated by this atom economic and operationally simple method. A total of 15 compounds were synthesized under this mild reaction condition. Based on a series of experimental results the author proposed a mechanistic pathway for this transformation which involved the oxidation of styrene 57a to the corresponding radical (Int-12) under the influence of I_2_/TBHP through a radical mechanism as depicted in Scheme 17. Iodination of Int-12 by I_2_/TBHP afforded the key intermediate Int-13 which undergo Kornblum oxidation to produce phenylglyoxal Int-14. Subsequent treatment with diamine 17, the intermediate Int-14 furnished intermediate Int-15, which on cyclization followed by oxidation provides quinoxaline 58a.

**Scheme 18:**
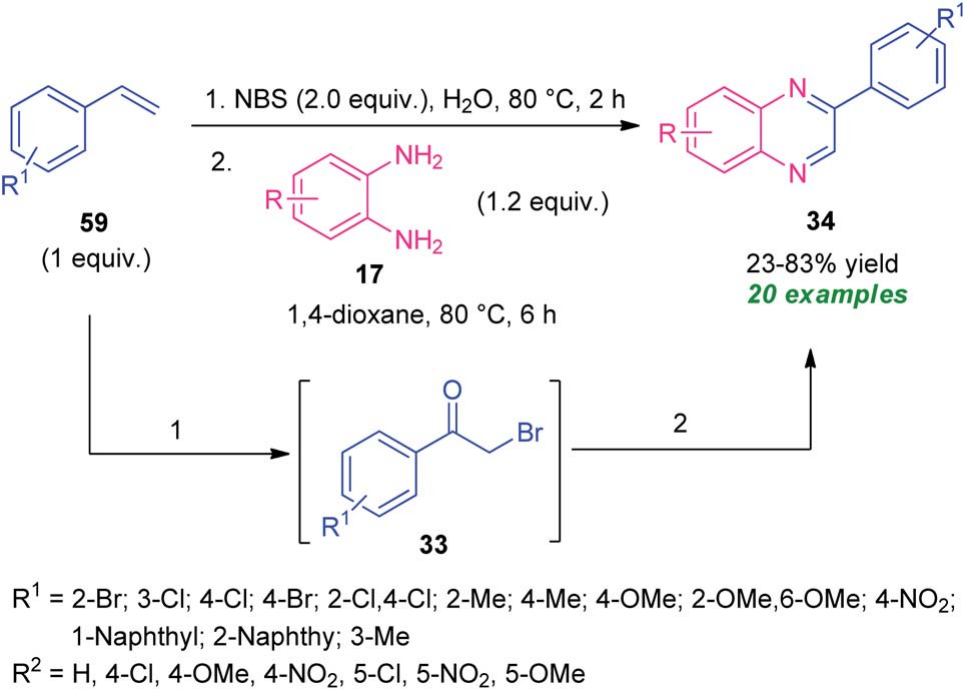
NBS-promoted one-pot two-step green synthesis of quinoxalines 34.

A practical one-pot two-step NBS-promoted efficient synthesis of quinoxalines from the reaction of substituted alkenes 59 and *o*-phenylenediamines 17 was established (Scheme 18).^87^ The synthesis involves the initial reaction of alkenes 59 with NBS in aqueous media to deliver the phenacyl bromide 33. Subsequent condensation of 33 with diamine 17.

In presence of 1,4-dioxane formed the quinoxaline derivatives 34 in 23–83% yields. A wide variety of substituents present on the alkenes ring smoothly anticipated the reaction under this metal-free approach. Broad functional group tolerance, utilization of inexpensive NBS as bromine source as well as oxidant, water as green solvent, a high level of yield make this approach synthetically as well as environmentally viable.

### 3.6 From alkynes

**Scheme 19:**
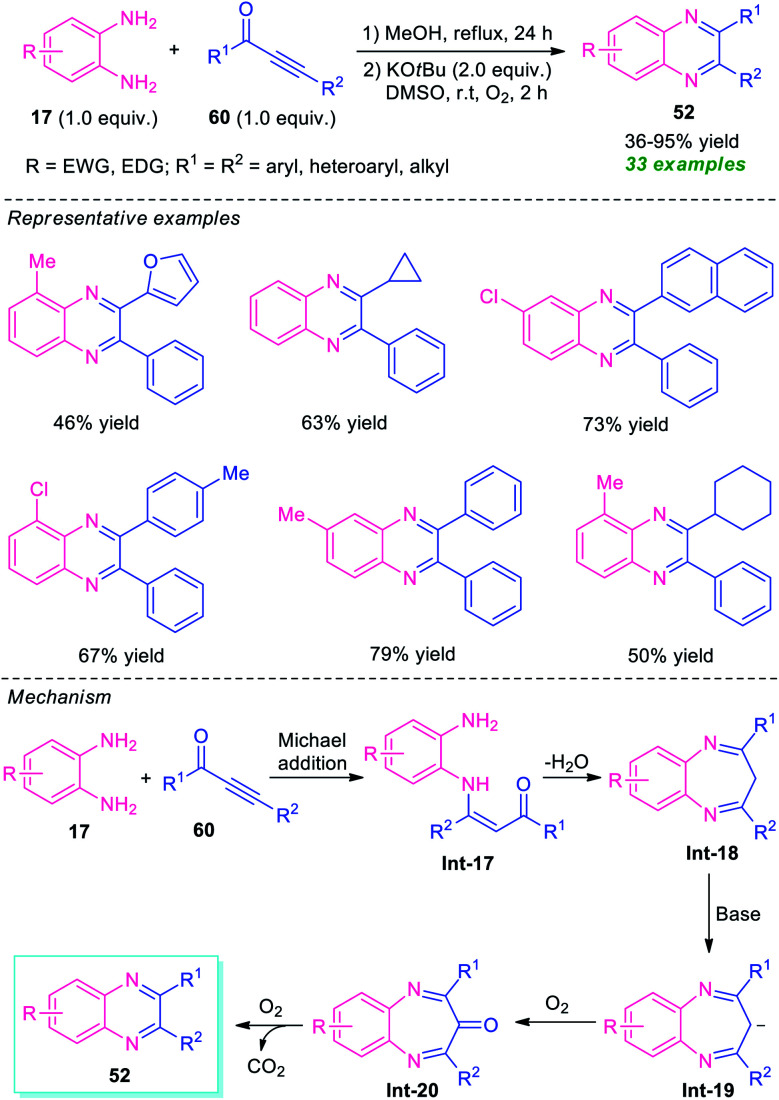
Metal-free one-pot synthesis of quinoxalines *via* C–α-CH_2_-extrusion process.

In 2016, Cui and his group disclosed a highly convenient one-pot approach for the regiospecific synthesis of quinoxalines 52 from readily available ynones 60 and *o*-phenylenediamines 17 under metal-free conditions (Scheme 19).^88^ The reaction proceeded through an intermolecular Michael addition, followed by dehydration and subsequent base-promoted C–α-CH_2_-extrusion process. A total of 33 quinoxaline derivatives were synthesized by this strategy in 36–95% yields. Various aryl, heteroaryl, and alkyl-substituted ynones were found to be suitable substrates for this reaction. However, when asymmetrical *o*-phenylenediamine and ynones were employed in the reaction, the products were formed in moderate to good yield with excellent regioselectivity (>20 : 1). The exploitation of O_2_ as the environmentally benign oxidant, formation of H_2_O and CO_2_ as only the by-products directed this approach toward more environmentally friendly and sustainable.

The proposed mechanism for this C–α-CH_2_-extrusion process involves the initial Michael addition of *o*-phenylenediamines 17 and ynones 60 to formed intermediate Int-17 regioselectively that can then undergo dehydration as well as condensation to give Int-18. Subsequent deprotonation of Int-18 by KO*t*Bu results in the formation of anion intermediate Int-19 which gets easily oxidized to Int-20 by O_2_. The final decarbonylation of Int-20 under O_2_ afforded the products 52.

**Scheme 20:**
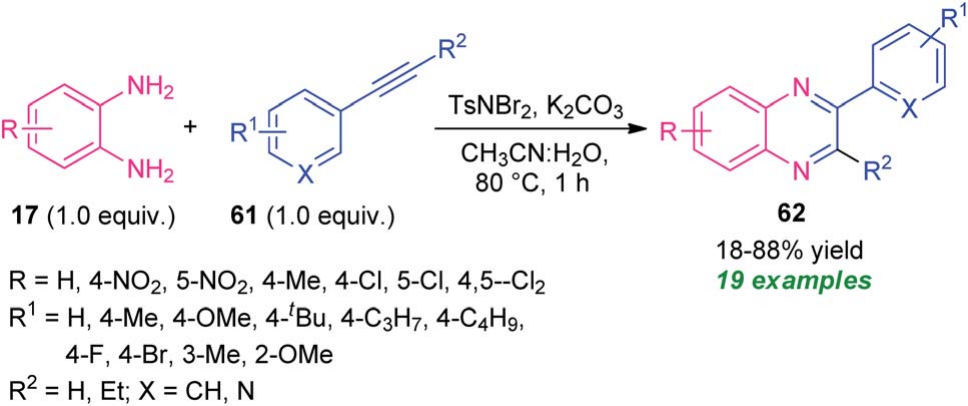
Metal-free domino protocol for the synthesis of quinoxalines *via* oxidative cyclization.

A metal-free cascade process toward the synthesis of diverse quinoxaline derivatives 61 from alkynes 60 has been developed by Hazarika and Phukan in 2017 (Scheme 20).^89^ Treatment of alkynes 61 with TsNBr_2_ (*N*,*N*-dibromo-*p*-toluene sulfonamide) in an aqueous medium first generated the α,α-dibromoketone intermediate which on reaction with 17 under influence of base deliver the desired products 62 in 18–88% yields within one hour. The scope of the reaction was found to be viable to a wide variety of substituents on the phenyl ring of the alkynes as well as diamines. However, the low yield of the products for the reaction with nitro- and chloro-substituted diamines represents a limitation of this approach.

**Scheme 21:**
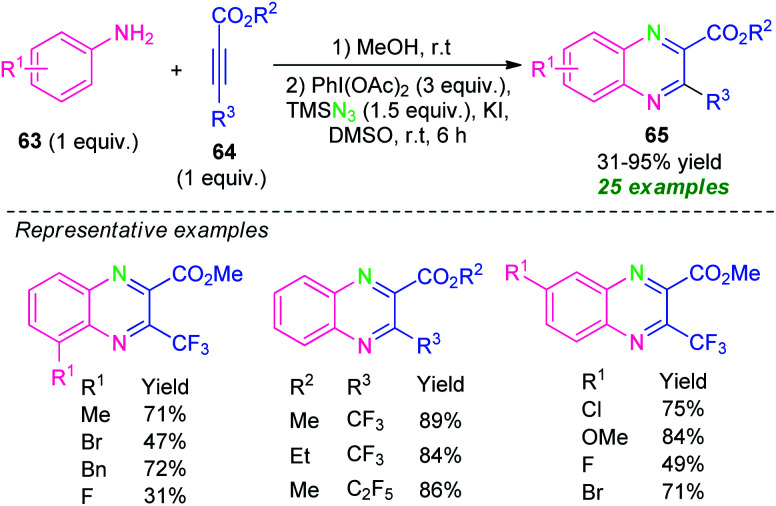
Tandem Michael addition/azidation/cycloamination sequence toward the synthesis of quinoxalines.

In 2018, Zhang, Shen, Cao, and their group demonstrated a Michael addition initiated tandem azidation and cycloamination reaction for the construction of diverse fluoroalkylated quinoxalines (Scheme 21).^90^ Under metal-free conditions, the corresponding quinoxalines derivatives 65 derived from anilines 63 and fluoroalkylated alkynes 64 and TMSN_3_ as the nitrogen source, were obtained in 31–95% yields. The reactions with *ortho*-substituted anilines were found to lead to a decrease in the yield of the products as compared to *para*-substituted anilines. Alternatively, when *meta*-substituted anilines were employed as the substrates, two regioisomers has been formed in moderate to excellent yield. In this reaction, the utilization of KI as an additive provides the formation of iodine(i) species by reacting with PhI(OAc)_2_ and thereby promoting the azidation and cycloamination sequence toward the synthesis of the final products.

### 3.7 From enaminones

Enaminones are an important class of widely investigated organic compounds in organic chemistry. Due to the highly reactive nature and easy availability, a great variety of organic compounds have been synthesized by making the utilization of enaminones as key building blocks.^91^

**Scheme 22:**
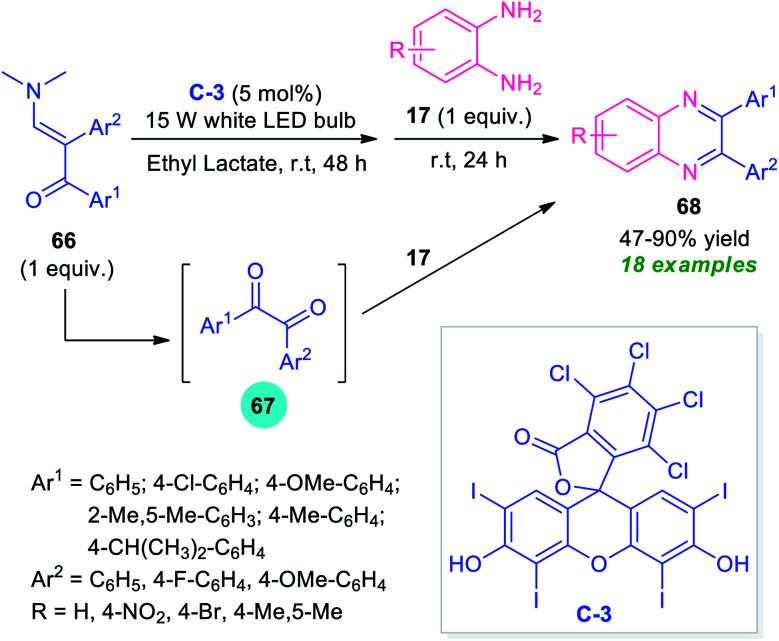
Visible-light induced photocatalytic cleavage of C=C of enaminones to access quinoxalines.

By considering this key importance and taking advantage of the high photocatalytic activity of Rose Bengal C-3, Wan, Wen, and co-workers disclosed a one-pot procedure for the synthesis of quinoxalines from enaminones 66 (Scheme 22).^92^ With the help of 5 mol% of C-3 as the photocatalyst, the visible light-mediated reaction of enaminones 66 afforded the corresponding products 68 in 47–90% yields. The overall process starts with the cleavage of C=C of enaminones 66 to furnish the adduct 67 which reacts *in situ* with *o*-phenylenediamines 17 under the ambient condition to form the resultant products 68. Broad functional group tolerances, easy accessibility of the starting material, operational simplicity are some of the key advantages of this approach. The low yield of products for the reaction with *o*-phenylenediamines bearing electron-withdrawing group constitutes a major drawback of this method.

### 3.8 From isonitriles

**Scheme 23:**
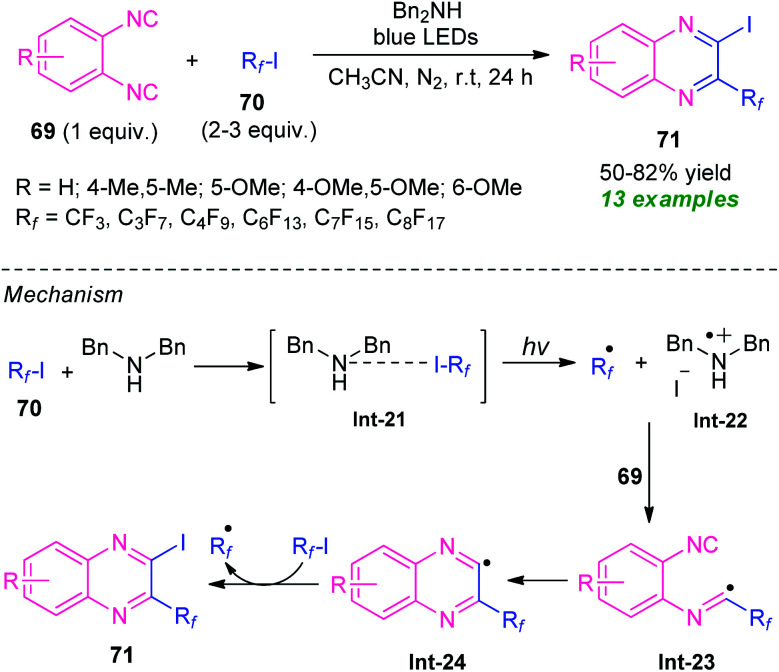
Halogen-bond-promoted construction of various quinoxaline derivatives under visible light irradiation.

In 2016, the research group of Ma and Yu developed a visible light-assisted halogen-bond-promoted strategy for the synthesis of quinoxaline derivatives through the double radical isocyanide insertion of *o*-diisocyanoarenes 69 with perfluoroalkyl iodides 70 (Scheme 23).^93^ By using this strategy, a series of diverse 2-fluoroalkylated 3-iodoquinoxalines 71 were synthesized in moderate to high yield at room temperature. The overall process can initiate through the formation of halogen-bond adduct Int-21 which under the influence of visible light deliver the fluoroalkyl radical R_f_˙, Bn_2_NH^+^˙, and iodide anion *via* a single electron transfer (SET) process. In this reaction, perfluoroalkyl iodides serve as halogen-bond donors and Bn_2_NH as halogen-bond acceptors. The addition of fluoroalkyl radical R_f_˙ to *o*-diisocyanoarenes 69, followed by radical cyclization produces the radical intermediate Int-24 which then abstracts an iodine atom from 70 to yield the final products 71.

**Scheme 24:**
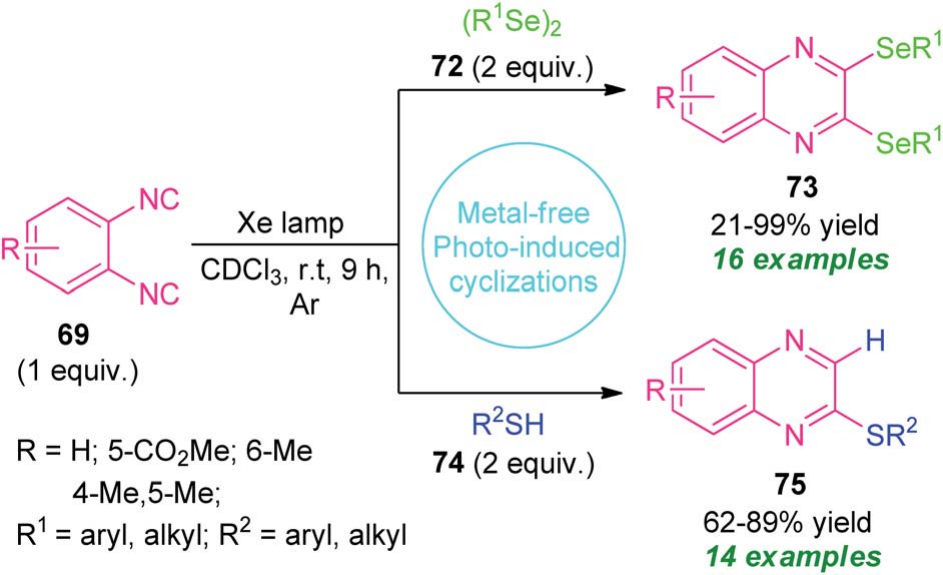
Visible light irradiated construction of different quinoxalines derivatives under metal-free conditions.

Recently, a metal- and additive-free cyclization sequence under visible-light irradiation has been established for the synthesis of a vast array of quinoxaline derivatives from *o*-diisocyanoarenes 69 and organic diselenides or thiols (Scheme 24).^94^ Initial optimization of the reaction condition revealed that utilization of Xe lamp as a light source in presence of CDCl_3_ as the solvent provides high activity and reactivity of the reaction as compared to high-pressure Hg lamp. By using this condition, a series of 2,3-bis-(selanyl)quinoxalines 73 derived from 69 and 72, were obtained in 21–99% yields. Not only the aryl-substituted selenides but also alkyl-substituted selenides were reacted efficiently under this condition.

Encourage by this result, they further attempted the reaction with 69 and 74 under the same reaction condition. Delightfully, a total of fourteen 2-thiolated quinoxaline compounds 75 were synthesized in moderate to high yield. Unlike diselenides, the reaction condition also tolerates several alkyl and aryl-substituted thiols. But unfortunately, thiols bearing unprotected alcohol groups, amines, and (^i^Pr)_3_SiSH failed to yield any products which represents a major limitation of this approach (Scheme 24).

### 3.9 From oximes

**Scheme 25:**
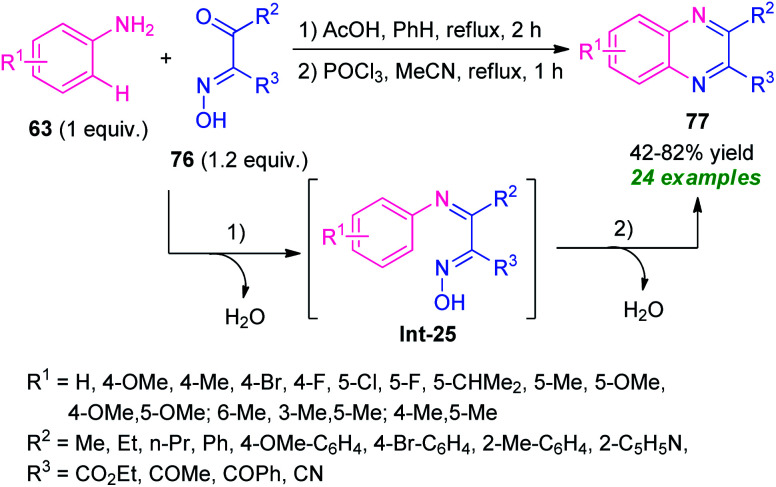
One-pot tandem cyclization/metal-free *N*-arylation toward the synthesis of functionalized quinoxalines.

A tandem one-pot protocol for the construction of functionalized quinoxalines from easily accessible arylamines 63 and β-keto oximes 76 has been developed by Pan, Mo, Su, and co-workers in 2017 (Scheme 25).^95^ The reaction sequence involves the initial condensation of amines 63 with oximes 76 as the nitrogen source and subsequent metal-free *N*-arylation of key intermediate Int-25 under Lewis acid catalysis was found to lead to the desired products 77 in 42–82% yields. The effectiveness of the developed protocol was established by its applicability in the gram-scale synthesis and other useful transformations. A wide range of substrate scopes, exploitation of the synthesized compounds in useful synthetic modifications, mild reaction conditions, water as only the by-products, systematic design of the metal-free sequence makes this protocol synthetically valuable and environmentally benign.

## 4. Synthesis of fused quinoxalines based on metal-free two-component reactions

### 4.1 Synthesis of pyrrolo/indolo[1,2-*a*]quinoxalines

**Fig. 3:**
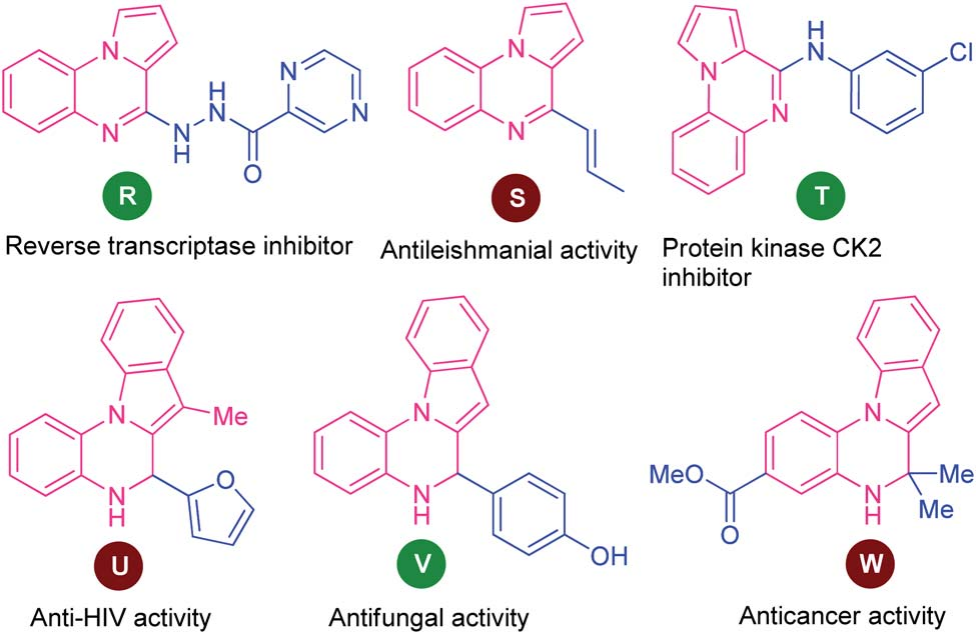
Biologically active various pyrrolo/indolo[1,2-*a*]quinoxalines derivatives.

Among various fused quinoxaline derivatives, pyrrolo/indolo[1,2-*a*]quinoxalines as the most important and well-established nitrogen-containing heterocycles play a vital role in medicinal and pharmaceutical chemistry due to their remarkable pharmacological profile.^96^ In particular, the presence of this moiety in many products found to exhibit reverse transcriptase inhibitor (R), antileishmanial activity (S), protein kinase CK2 inhibitor (T), anti-HIV activity (U), antifungal activity (V), and anticancer activity (W) (Fig. 3).^97^ Owing to these tremendous properties, significant efforts have been demonstrated toward their synthesis in the last decades.

#### 4.1.1 From carbonyl compounds

In the past decades, a vast array of procedures toward the rapid access of pyrrolo[1,2-*a*]quinoxaline motifs has been reported; among them, the Bischler–Na-pieralski reaction or modified Pictet–Spengler reaction and also one-pot reaction has widely been studied.^98^ However, most of this reaction suffers serious drawbacks due to the exploitation of transition-metal-catalysts, volatile, and toxic reagents alongside the low yield of the products. Therefore, there is a need for developing a high atom economic, environmentally friendly, and eco-compatible approach to pyrrolo[1,2-*a*]quinoxaline structure.

**Scheme 26:**
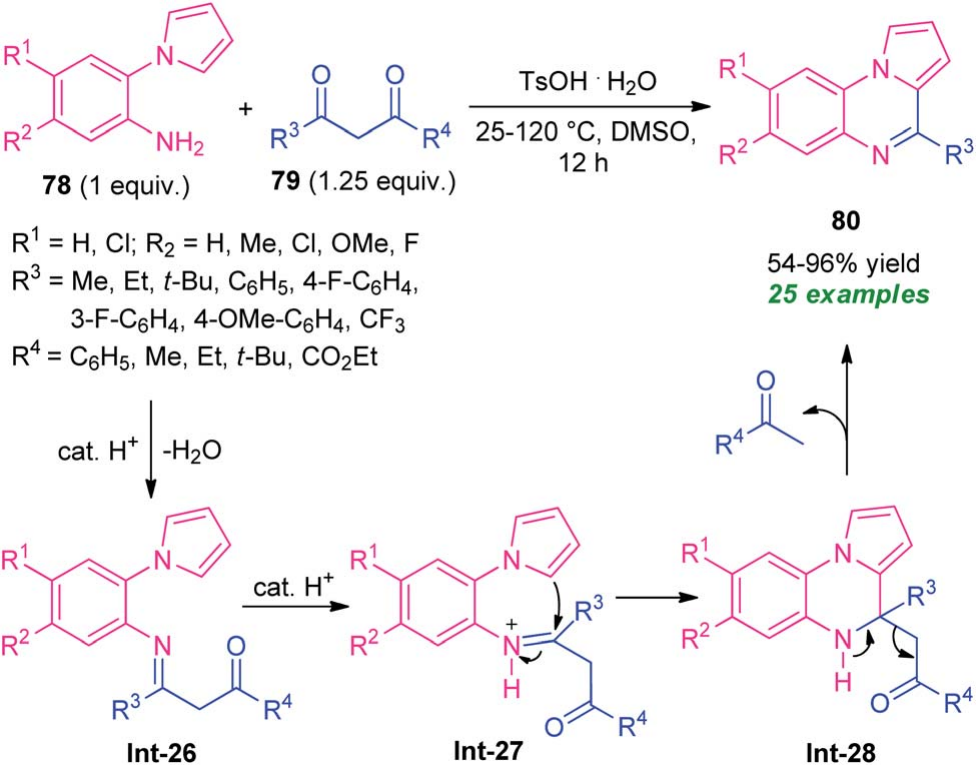
Brønsted acid-catalyzed one-pot domino synthesis of pyrrolo[1,2-*a*]quinoxalines.

In this perspective, Ma and co-workers in 2016, developed a highly efficient one-pot domino strategy by employing 2-(1*H*-pyrrol-1-yl)aniline 78 and 1,3-dicarbonyl compounds 79 as the easily available starting material (Scheme 26).^99^ With transition metal-free condition, a variety of pyrrolo[1,2-*a*]quinoxalines 80 in 54–96% yields has been synthesized utilizing TsOH·H_2_O as the organocatalyst. The reaction condition was found to be appropriate for both β-diketones 79 (R^3^ = aryl, alkyl; R^4^ = aryl, alkyl) and β-keto esters 79 (R^3^ = aryl, alkyl; R^4^ = OEt). However, the reaction with diketones 79 (R^3^ = R^4^ = *t*-Bu) failed to yield any product which represents the limitation of this approach.

The requirement of a longer reaction time also points toward the drawback of this method. This transformation can proceed *via* the initial formation of the imine intermediate Int-26, which then abstracts a proton from the catalyst to form Int-27. Subsequently, an intramolecular cyclization of intermediate Int-27 followed by cleavage of C–C bond in intermediate Int-28 form the final product 80.

**Scheme 27:**
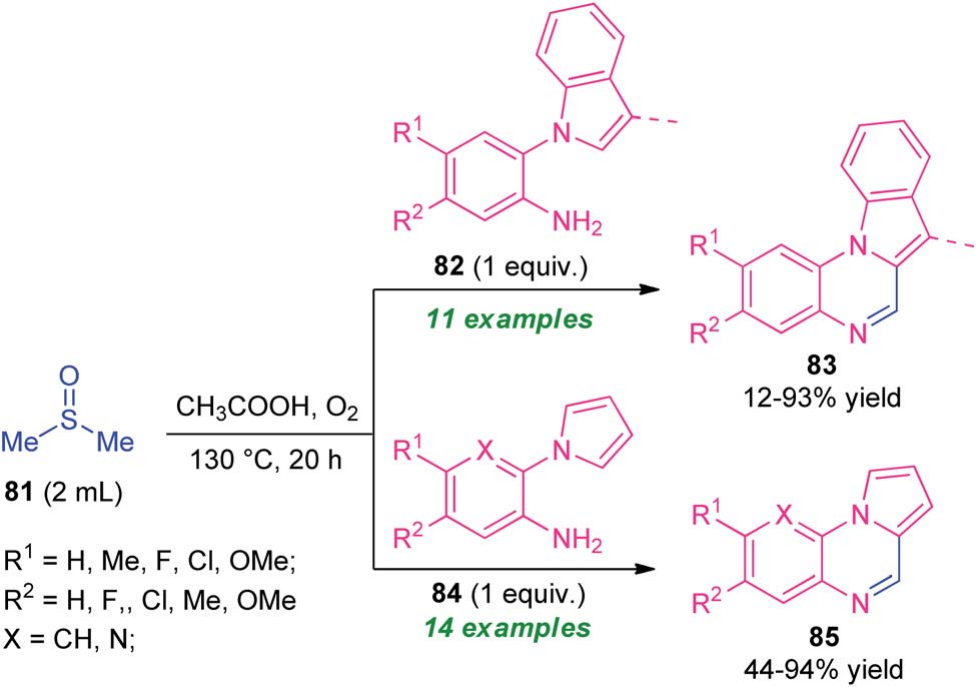
Versatile activity of DMSO in the synthesis of quinoxaline.

One year later, the same research group disclosed a novel one-pot green strategy for the synthesis of pyrrolo/indolo[1,2-*a*]quinoxalines by employing DMSO as reactant as well as the solvent system in metal-free condition (Scheme 27).^100^ With the help of AcOH acid as the catalyst, treatment of dimethyl sulfoxide 81 with 2-(1*H*-indol-1-yl)aniline 82 was found to take place at 120 °C to deliver indolo[1,2-*a*]quinoxalines 83 in 12–93% yields while using 2-(1*H*-pyrrol-1-yl)aniline 83 instead of 82 under the same reaction condition provided pyrrolo[1,2-*a*]quinoxalines 85 in 44–94% yields. A wide variety of electron-rich, as well as electron-poor substituents present on 82 and 84, were well tolerated by this condition. However, the low yield of the products 83 (12% yield) for the reaction with 2-(1*H*-indol-1-yl)aniline 82 (R^1^ = H, R^2^ = H; 3-Me is absent in indole ring)and no reaction for 3-methyl-2-(1*H*-pyrrol-1-yl)aniline 84 (R^1^ = R^2^ = H; X = C–Me), constitutes a major limitation of this approach. In addition, the strategy offers several advantages including mild reaction conditions, metal-free, utilization of oxygen as cheap and green oxidant, *etc.*

**Scheme 28:**
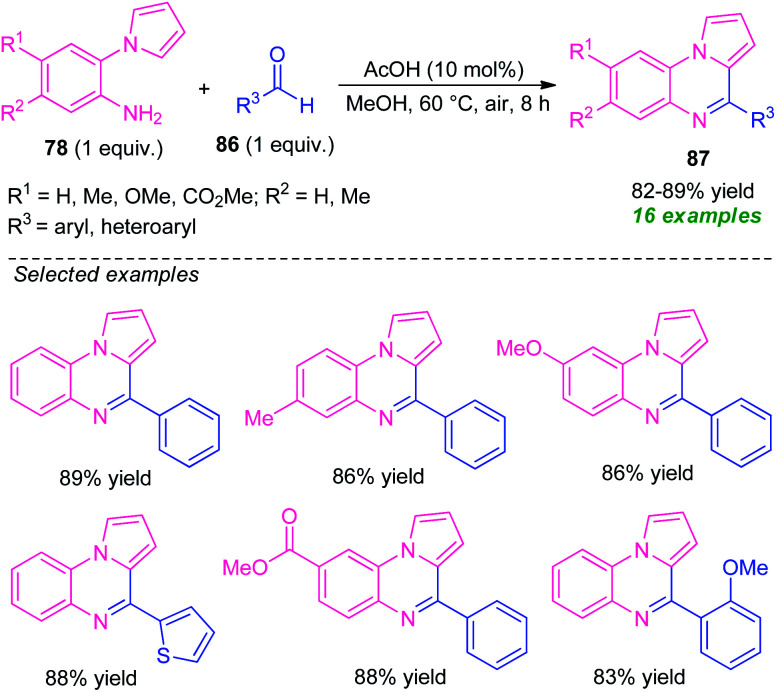
Acetic acid-catalyzed efficient synthesis of pyrrolo[1,2-*a*]quinoxalines through Pictet–Spengler reaction.

In 2019, Patel *et al.* reported an efficient acetic acid-catalyzed synthesis of pyrrolo[1,2-*a*]quinoxalines 87*via* a Pictet–Spengler type reaction of 2-(1*H*-pyrrol-1-yl)aniline 78 to aldehyde 86 (Scheme 28).^101^ With only 10 mol% of AcOH acid as the catalyst, a total of 16 compounds were synthesized in 82–89% yields. Pleasingly, various substituted and unsubstituted 2-(1*H*-pyrrol-1-yl)aniline 78 underwent the reaction smoothly to provide the desired product efficiently. While, the reaction with CF_3_ and CO_2_Me substituted 78 (R^1^ = H, R^2^ = CF_3_, CO_2_Me) failed to yield any product which draws a shortcoming of this approach. Similarly, aryl, as well as heteroaryl substituted aldehydes, were efficiently worked under this condition, except for electron-withdrawing substituents present on the *meta*-position of the aryl ring of aldehyde (R^3^ = 3-NO_2_-C_6_H_4_, 3-Cl-C_6_H_4_, 3-Br-C_6_H_4_) which affected the outcome of the reaction. Despite these limitations, the broad functional group tolerance, easily accessible starting material, are some advantages of this protocol. In addition, this reaction doesn't require any extra oxidant or additives, and the air was used as a green oxidant.

**Scheme 29:**
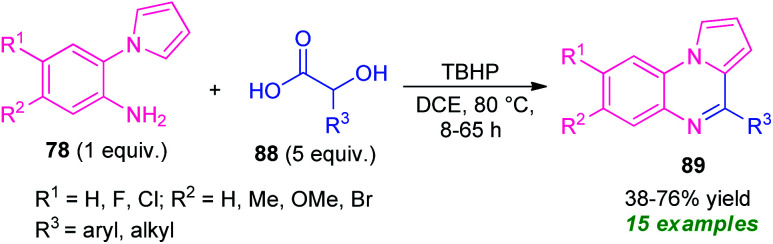
TBHP catalyzed metal-free synthesis of quinoxalines based on *in situ* generated aldehydes.

In 2020, Jung and their groups demonstrated α-hydroxy acid as an aldehyde surrogate for the construction of pyrrolo[1,2-*a*]quinoxalines based on a metal-free approach (Scheme 29).^102^ The synthesis involves the reaction of 2-(1*H*-pyrrol-1-yl)aniline 78 with α-hydroxy acid 88 in presence of TBHP (*tert*-butyl hydrogen peroxide) as the oxidant, leading to the desired products 89 in 38–76% yields. The key step behind this reaction is the *in situ* formation of aldehydes from α-hydroxy acid 88 and then condensation with 78, followed by intramolecular cyclization and subsequent oxidation step. Although diverse substitutions on the 2-(1*H*-pyrrol-1-yl)aniline ring and α-hydroxy acid could be tolerable, the low yield of the products, as well as the longer reaction time, constitutes a major drawback of this approach other than outstanding work.

#### 4.1.2 From alkenes and alkynes

**Scheme 30:**
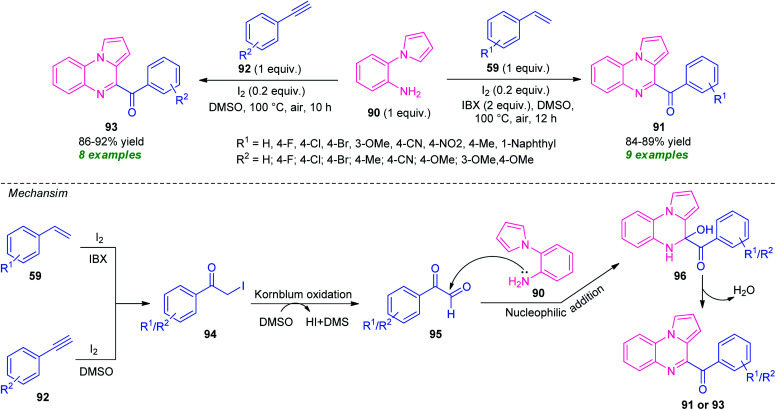
Metal-free diversity-oriented one-pot synthesis of fused quinoxaline derivatives from alkenes and alkynes.

In 2018, Reddy *et al.* demonstrated a highly efficient metal-free one-pot domino oxidative cyclization approach for the construction of several pyrrolo[1,2-*a*]quinoxaline derivatives from alkenes and alkynes (Scheme 30).^103^ With the help of I_2_ as the catalyst and 2-iodoxybenzoic acid (IBX) as the oxidant, the treatment of alkenes 59 with 2-(1*H*-pyrrol-1-yl)aniline 90 was found to take place in DMSO to deliver the desired products 91 in good yield. A variety of electron-donating and electron-withdrawing substituents present on the aryl ring of alkenes smoothly participated in this reaction. On the other hand, treatment of 90 with alkynes 92 in presence of a catalytic amount of I_2_ and DMSO as the solvent cum oxidant provides corresponding fused quinoxalines 93 in good to high yield. This reaction doesn't require IBX as an external oxidant. The methodology offers several advantages including mild reaction conditions, eco-compatible, *etc.* However, the requirement of a longer reaction time constitutes a limitation of this approach.

Initially, intermediate 94 was formed *in situ* either from 59 or from 92, which undergo Kornblum oxidation with DMSO to form an intermediate 95. A subsequent reaction of 95 with 90, followed by dehydration afforded the final products 91 or 93.

#### 4.1.3 From amines

In order to formulate an eco-compatible, atom-economic as well as environmentally friendly approach for the synthesis of pyrrolo/indolo[1,2-*a*]quinoxalines, several metal-free methods have been devised from readily available aryl/heteroaryl/alkyl amines and 1-(2-aminoaryl)pyrrole in the past decades.^104^

In 2015, Zhai *et al.*, and Jayaprakash *et al.*, independently reported two highly efficient metal-free one-pot strategies toward the synthesis of pyrrolo/indolo[1,2-*a*]quinoxalines 99 from 1-(2-aminoaryl)pyrrole/indole 97 and amines 98 (R^1^ = aryl) by employing molecular iodine as the catalyst as well as the promoter respectively. In 2019, direct one-pot access to several pyrrolo/indolo[1,2-*a*]quinoxalines 99 under metal-free green reaction conditions from 1-(2-amino/nitroaryl)pyrrole/indole 97 and amine 98 (R^1^ = aryl/alkyl/heteroaryl) were reported separately by Pardeshi, Chaskar, Patil and their groups and Wang *et al.* In all the cases, the corresponding products were formed in good to excellent yield (Table 2).^105–109^ Broad functional group tolerances, eco-friendly as well as environmentally friendly nature, operational simplicity, metal-free, additive-free, redox reagent-free, toxic-free, gram-scale synthesis, are some of the advantages of all of the reported methods. Although all the reported methodology has several significant advantages, they also suffer some limitations including longer reaction time, low yield of the products, higher energy source; products were isolated by conventional purification techniques that utilize volatile solvents and are also involved in the waste of solvents. Therefore, attention has also needed to be paid to these reported methodologies, to solve the concerns raised.

**Table 2:** One-pot reaction of 1-(2-aminoaryl)pyrrole/indole 97 with aryl amine 98 to access pyrrolo/indolo[1,2-*a*]quinoxalines 99 under metal-free conditions

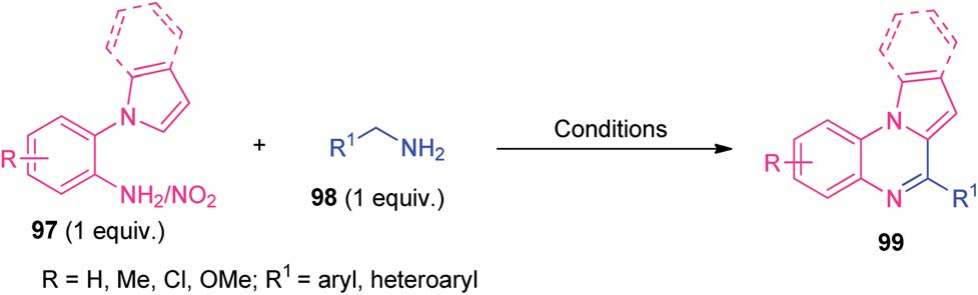									
Entry	Catalyst/promoter	Loading	Oxidant	Solvent	Temperature	Time (h)	Examples	Yield (%)	Ref.
1	I_2_	5 mol%	O_2_	*o*-Xylene	140 °C	12	27	79–95	105
2	I_2_	2 equiv.	—	MeCN	80 °C	5–8	22	82–95	106
3	—	—	K_2_S_2_O_8_	PEG-400	r.t	8	25	65–92	107
4	—	—	1,2-DNB^a^	Diglyme	130 °C	9	25	62–89	108
9	Ac^b^	15 mol%	—	H_2_O	140 °C	20	11	41–99	109

a 1,2-Dinitrobenzene.

b Activated carbon.

**Scheme 31:**
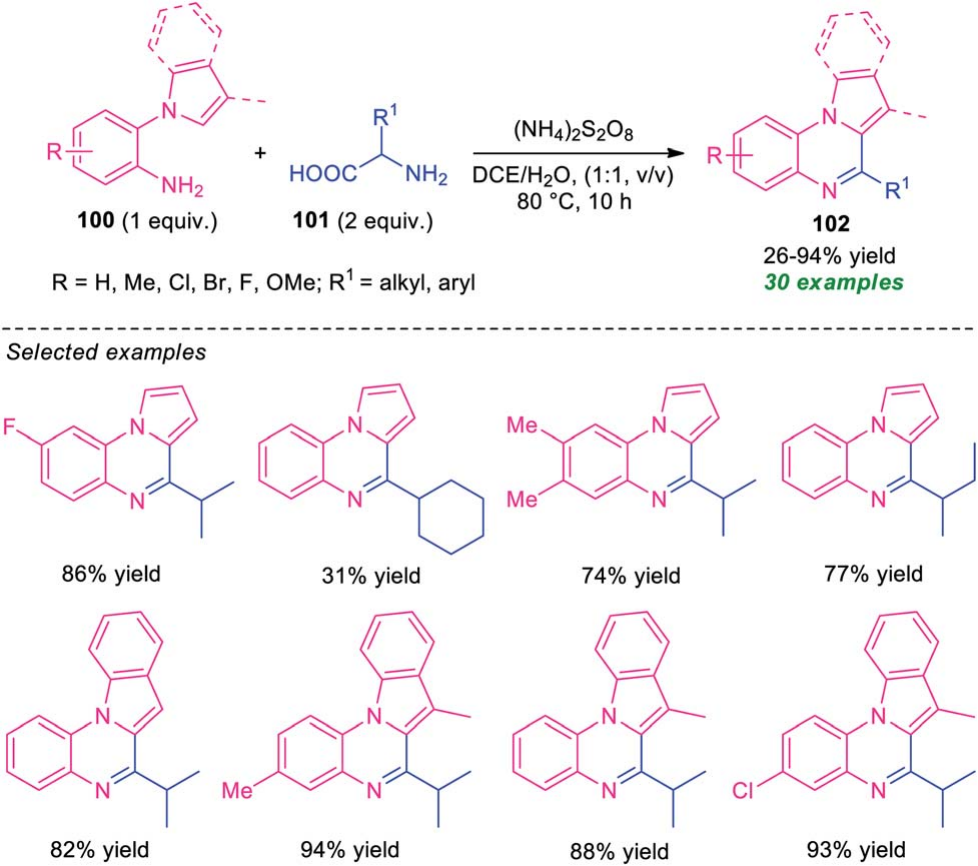
Transition-metal-free synthesis of fused quinoxalines from α-amino acid.

Another successful example for the synthesis of fused quinoxalines from amines under transition-metal-free conditions has been realized by Ma and co-workers (Scheme 31).^110^ With the help of (NH_4_)_2_S_2_O_8_ as the oxidant, the pyrrolo/indolo[1,2-*a*]quinoxaline products 102, derived from 1-(2-aminoaryl)pyrrole/indole 100 and α-amino acid 101, were obtained in poor to high yield. The optimized reaction conditions were found to be very compatible for a wide range of substituted 1-(2-aminoaryl)pyrrole/indole 100 possessing electron-deficient and electron-rich groups as well as for a variety of short- and long-chain amino acids. However, the reaction with methyl, *tert*-butyl, and cyclohexyl substituted amino acids (R^1^ = Me, *t*-Bu, cyclohexyl) gave only 26%, trace, and 31% yield of the products respectively. This is presumably due to involved steric hindrance that affects the outcome of this approach. Therefore, the development of a novel protocol that provides a clean pathway for expanding the hindered substrates scopes is highly desired.

### 4.2 Synthesis of imidazo[1,5-*a*]- and imidazo[1,2-*a*]quinoxalines

Unlike pyrrolo/indolo[1,2-*a*]quinoxalines, the derivatives of imidazoquinoxalines including imidazo[1,5-*a*]quinoxalines and imidazo[1,2-*a*]quinoxalines have extensively been studied and attracted considerable attention in medicinal and organic chemistry due to their tremendous biological activity such as anticancer activity, antimicrobial activity, antiallergenic activity, anticonvulsant activity, and kinase inhibitory activity.^111^ Because of this importance, significant efforts have been made for their synthesis.^112^

**Scheme 32:**
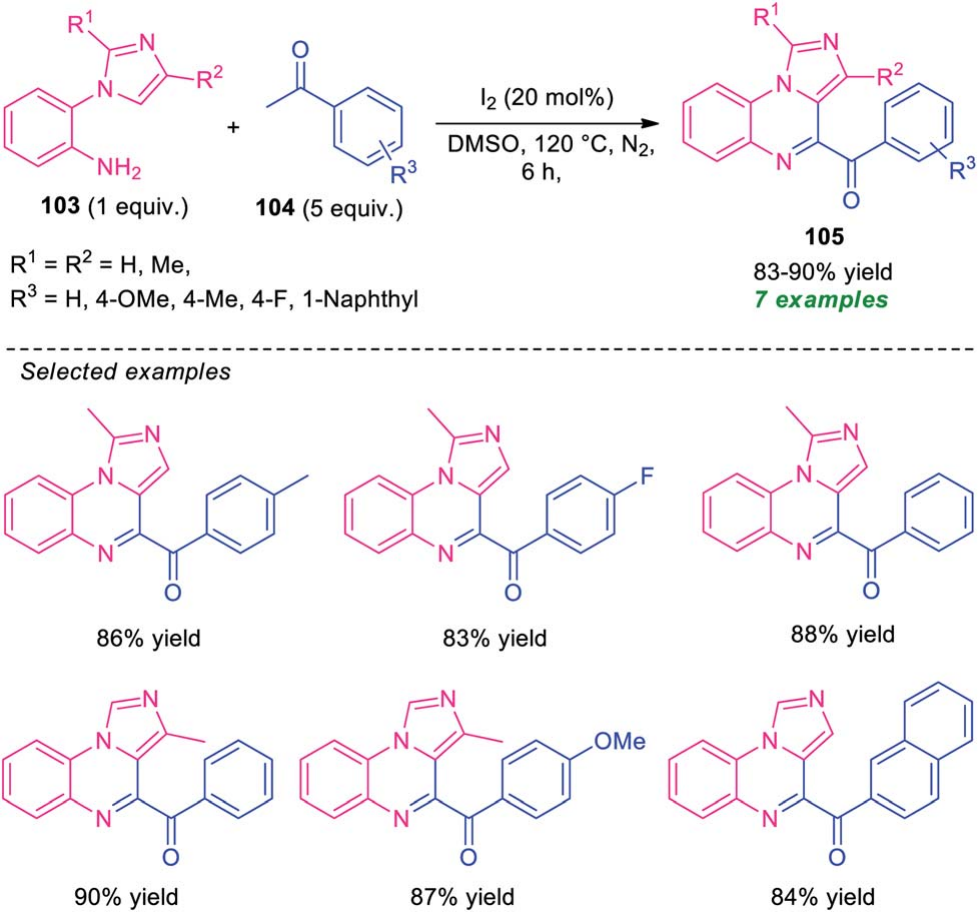
Metal-free iodine catalyzed one-pot synthesis of imidazo[1,5-*a*]quinoxalines 105.

In 2015, Ma and co-workers developed a metal-free one-pot cascade coupling process for the construction of imidazo[1,5-*a*]quinoxalines 105 (Scheme 32).^113^ By using 20 mol% of molecular iodine as the catalyst and DMSO as the oxidant as well as solvent, the reaction of 2-(1*H*-imidazol-1-yl)aniline 103 and ketones 104 were found to proceed smoothly under nitrogen atmosphere to afford the desired products 105 in good to high yield. Aryl-substituted ketones as well as substituted 2-(1*H*-imidazol-1-yl)aniline efficiently participated in the reaction under this condition. The advantages of this approach included mild reaction conditions, eco-compatible, readily accessible starting material, *etc.* However, the low substrate scopes alongside high catalyst loading, long reaction time constitutes a major limitation of this approach and needs ample attention to extended the substrate scopes of this approach.

**Scheme 33:**
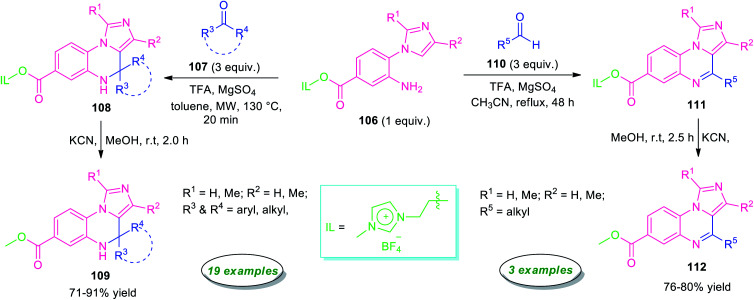
Regioselective metal-free synthesis of imidazo[1,5-*a*]quinoxalines from ionic liquid supported 2-(1*H*-imidazol-1-yl)aniline.

Highly regioselective synthesis of imidazo[1,5-*a*]quinoxalines *via* an unconventional Pictet–Spengler reaction was reported by Chung, Sun, and their group in 2016 (Scheme 33).^114^ Treatment of ionic liquid supported 2-(1*H*-imidazol-1-yl)aniline 106 with ketones 107 in presence of TFA under microwave condition at 130 °C, afforded ionic liquid immobilized imidazo[1,5-*a*]quinoxalines 108 within 20 minutes. Subsequently, cleavage of ionic liquid from 108 was accomplished by KCN in MeOH at room temperature to furnish the final products 109 in moderate to high yield. The reaction condition was found to be compatible with a broad range of aryl, heteroaryl, and alkyl-substituted ketones. However, when the same reaction was carried out with aryl aldehydes instead of ketones, aryl derivatives of imidazo[1,5-*a*]quinoxalines bearing ionic liquids were formed, but on treating with KCN for removal of ionic liquids, only cleavage of C(3*a*)-C(4) bond was observed rather than getting the final products. Pleasingly, modify the reaction condition from microwave to reflux in CH_3_CN rather than toluene, aliphatic aldehydes found to underwent the reaction smoothly providing the final imidazo[1,5-*a*]quinoxalines 112 in 76–80% yields. However, narrow substrate scopes represent a limitation of this approach.

**Scheme 34:**
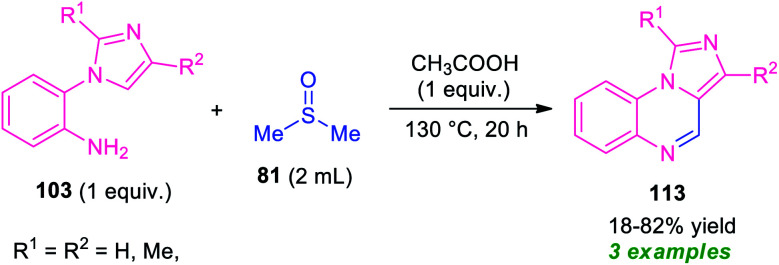
Synthesis of imidazo[1,5-*a*]quinoxalines 113 by using DMSO as solvent cum reagent in metal-free condition.

As a continuation of their outstanding work on the synthesis of pyrrolo/indolo[1,2-*a*]quinoxalines described in Scheme 27, Xie and co-workers also disclosed the synthesis of imidazo[1,5-*a*]quinoxalines 113 by employing metal-free reaction condition (Scheme 34).^100^ With the help of DMSO as the solvent cum reagent, the corresponding imidazo[1,5-*a*]quinoxalines 113 derived from 1-(2-aminophenyl)imidazole 103, has been achieved in 18–82% yields. However, narrow substrates scopes and low yield of the products call for further development of this approach.

**Scheme 35:**
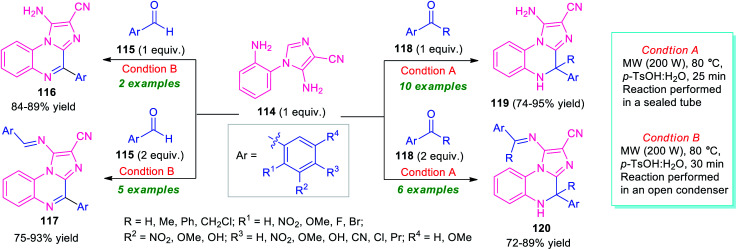
Metal-free microwave-assisted one-pot preparation of library of imidazo[1,2-*a*]quinoxalines.

In 2018, Kumar *et al.* introduced the microwave technique as a powerful green energy source for the synthesis of diverse imidazo[1,2-*a*]quinoxalines *via* a *6-endo-trig* cyclization of 5-amino-1-(2-aminophenyl)-imidazole-4-carbonitriles 114 with carbonyl compounds (Scheme 35).^115^ By using *p*-TsOH as a metal-free catalyst, treatment of the equimolar amount of aldehyde 115 with 114 performed in an open condenser under microwave heating at 80 °C, provides imidazo[1,2-*a*]quinoxalines 116 in 84–89% yields. While the same reaction, when carried out with 2 equivalents of 115 and 1 equivalent of 114, the products 117 were formed in 75–93% yields. Conversely, the reaction of an equimolar amount of ketone 118 and 114 performed in a sealed tube under microwave irradiation, afforded dihydroimidazo[1,2-*a*]quinoxalines 119 in 74–95% yields, whereas, employing 2 equivalents of 118 under the same reaction condition deliver products 120 in moderate to good yield. The utilization of microwave techniques not only reduces the reaction time in both cases but also provides a clean pathway for these reactions and improves the yield of the products as compared to the conventional method.

**Scheme 36:**
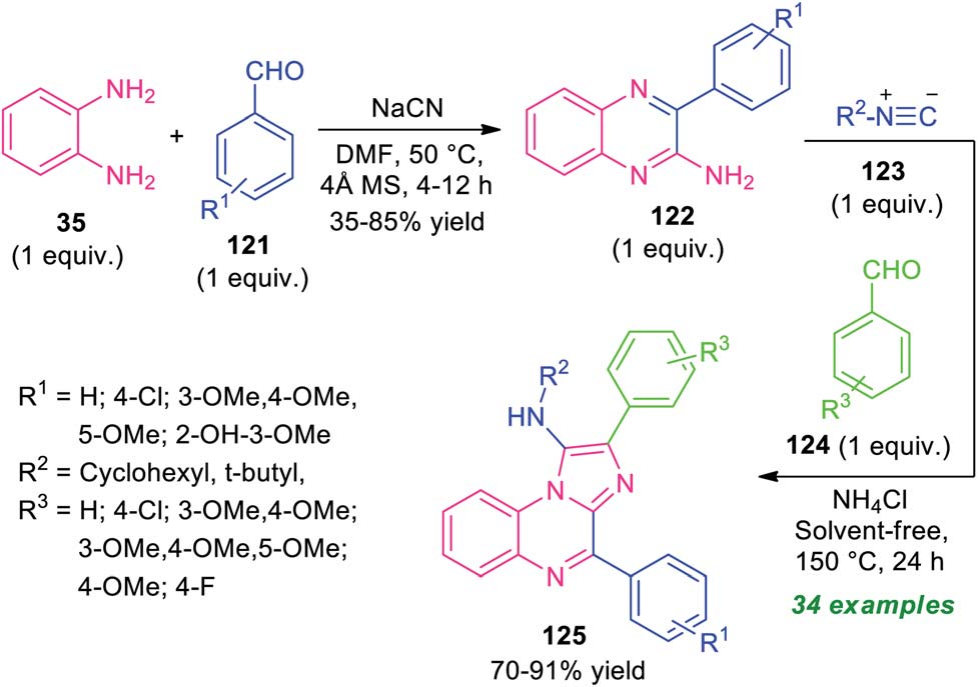
Two-step synthesis of imidazo[1,2-*a*]quinoxalines in metal-free condition.

A step-wise synthetic route for imidazo[1,2-*a*]quinoxalines 125 has been realized recently by Amini and his groups (Scheme 36).^116^ In the initial step, the reaction of *o*-phenylenediamine 35 with aldehydes 121 was carried out by using NaCN as a catalyst in DMF at 50 °C. This step requires 4 Å molecular sieves as the additives. The resulting amino-substituted quinoxalines 122 were formed in 35–85% yields within 4–12 hours. The second step involves a multicomponent reaction of an equimolar amount of 122 with isocyanides 123 and aldehydes 124 using ammonium chloride as the catalyst in solvent-free condition at 150 °C for 24 hours. The corresponding imidazo[1,2-*a*]qunioxaline derivatives 125 were formed in moderate to good yield. A variety of aryl aldehydes possessing different electron-withdrawing as well as electron-donating substituents well worked by this stepwise protocol. Broad functional group tolerances, solvent-free conditions, metal-free nature, a high level of yield are some of the salient features of this approach.

### 4.3 Synthesis of other fused quinoxalines

**Scheme 37:**
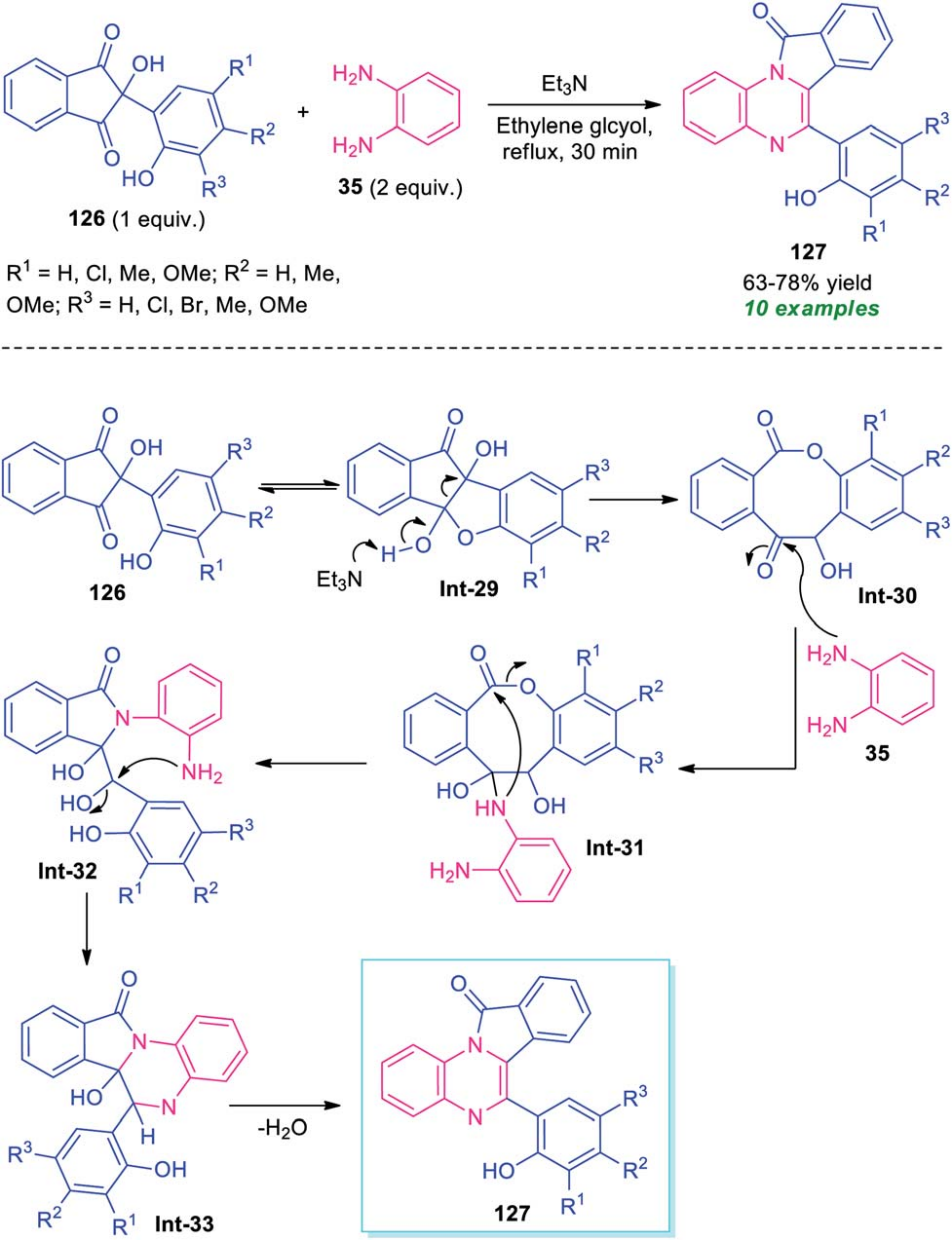
Metal-free base promoted tandem cyclization approach toward the rapid access of fused quinoxalines.

A metal-free tandem cyclization approach to access other fused quinoxalines, namely isoindolo[2,1-*a*]quinoxalines 127 has been demonstrated by Das *et al.*, in 2019 (Scheme 37).^117^ To find out the optimum condition, the reaction of ninhydrin 126 bearing phenolic adducts, with *o*-phenylenediamine 35 was initially performed in the presence of different bases including Et_3_N, piperazine, pyridine, Et_2_NH, and solvent system like ethylene glycol, water, MeOH, EtOH, glycerol. From these experiments, it was concluded that the utilization of Et_3_N in ethylene glycol provides easy access to the products within a very short reaction time. The optimum reaction condition was found to be compatible for a wide range of substituted phenolic ninhydrin adducts 126 that smoothly underwent the reaction to deliver the isoindolo[2,1-*a*]quinoxaline products 127 in 63–78% yields. Most of the synthesized compounds were found to have a fluorescent activity which remarks outstanding achievements of this work.

A mechanistic investigation suggests the initial formation of 8-membered lactone intermediate Int-30 from Et_3_N catalyzed cleavage of C–C bond of Int-29. The lactone Int-30 then experiences nucleophilic attack from –NH of 35 to form an intermediate Int-31, which provides intermediate Int-32*via* an intramolecular nucleophilic attack of –NH to C=O moiety and subsequent breaking of C–O bond. Consequently, the intramolecular cyclization of (Int-30), followed by elimination of water leads to the final products 127 (Scheme 37).^117^

## 5. Synthesis of simple quinoxalines based on metal-free multicomponent reactions

The development of an efficient concise route that provides easy access to highly functionalized and value-added compounds by fulfilling the criteria of green and sustainable chemistry has emerged as a most significant goal and formidable challenge for the synthetic chemist. Compared to synthetic step-wise process which deals with a number of limitations in the product development as well as in consistent with green chemistry principles, multicomponent reactions (MCRs) has recently come to receive widespread global acceptances as the most valuable choice of synthetic routes in industry and academia.^118–121^ Multicomponent reactions (MCRs) provide a clean and safe platform for multiple-bond forming events to occur in a single one-pot operation from more than two starting materials without separation and purification of the intermediates. Consequently, this reaction increases the product development rates by shortening the reaction steps, and also by reducing waste formation, utilizing inexpensive easily accessible reagents and reactants, *etc.*

**Scheme 38:**
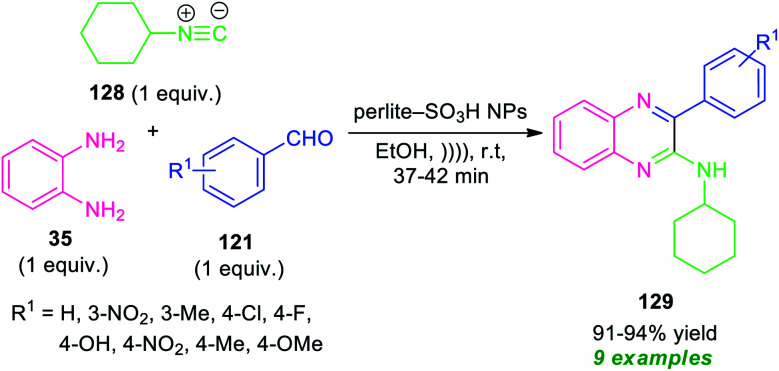
Ultrasound-assisted isocyanide-based multicomponent approach for the synthesis of quinoxalines 129.

As a consequence of these tremendous academic, economic and ecological interests, an isocyanide-based multicomponent approach^122^ for the synthesis of highly functionalized quinoxalines has been realized by Rouhani and Ramazani in 2018 (Scheme 38).^123^

By using perlite-SO_3_H nanoparticles as the catalyst, the three-component reaction of *o*-phenylenediamine 35, aldehydes 121, and cyclohexyl isocyanide 128 under ultrasonic irradiation were found to take place smoothly within a very short reaction time to provide the easy access of corresponding quinoxaline derivatives 129 in 91–94% yields. A variety of electron-withdrawing, as well as electron-donating groups present on the aryl ring of aldehydes, were well tolerated by this methodology. The exploitation of ultrasonic technology provides a clean synthesis of the desired quinoxaline products within a very short reaction time as compared to the conventional method which takes lots of time for the completion of the reaction.

**Scheme 39:**
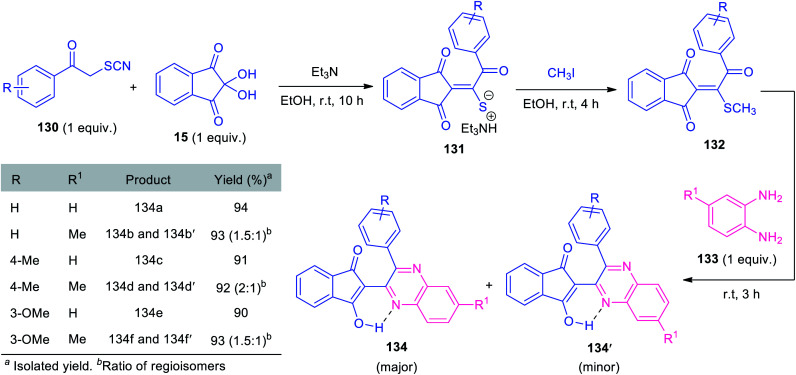
Application of triethylammonium thiolate salts as efficient reagents for the multicomponent synthesis of quinoxalines 134.

In 2019, Alizadeh and co-workers synthesized triethylammonium thiolate salts 131 based on a formal [3 + 2] cycloaddition, followed by subsequent ring-opening reaction of phenacyl thiocyanate 130 with ninhydrin 15 and has been demonstrated as an efficient reagent for the synthesis of quinoxalines. Under metal-free reaction conditions, a one-pot sequential three-component reaction of triethylammonium thiolate salts 131, with methyl iodide and *o-*phenylenediamines 133 was found to proceed smoothly to deliver several quinoxalines 134 bearing indandione framework, in high yields (Scheme 39).^124^ Compound 131 containing various electron-rich groups efficiently participated in this reaction. Similarly, simple *o*-phenylenediamine 133a (R^1^ = H) selectively afforded 134 as the major products. However, when methyl-substituted diamine 133b (R^1^ = Me) was employed as the substrates, two regioisomeric products (134 and 134′) were achieved. Despite the remarkable advantages including mild condition, high yield, operational simplicity, and eco-benign, the limited substrate scope represents a limitation of this approach and calls for further developments.

## 6. Synthesis of fused quinoxalines based on metal-free multicomponent reactions

**Scheme 40:**
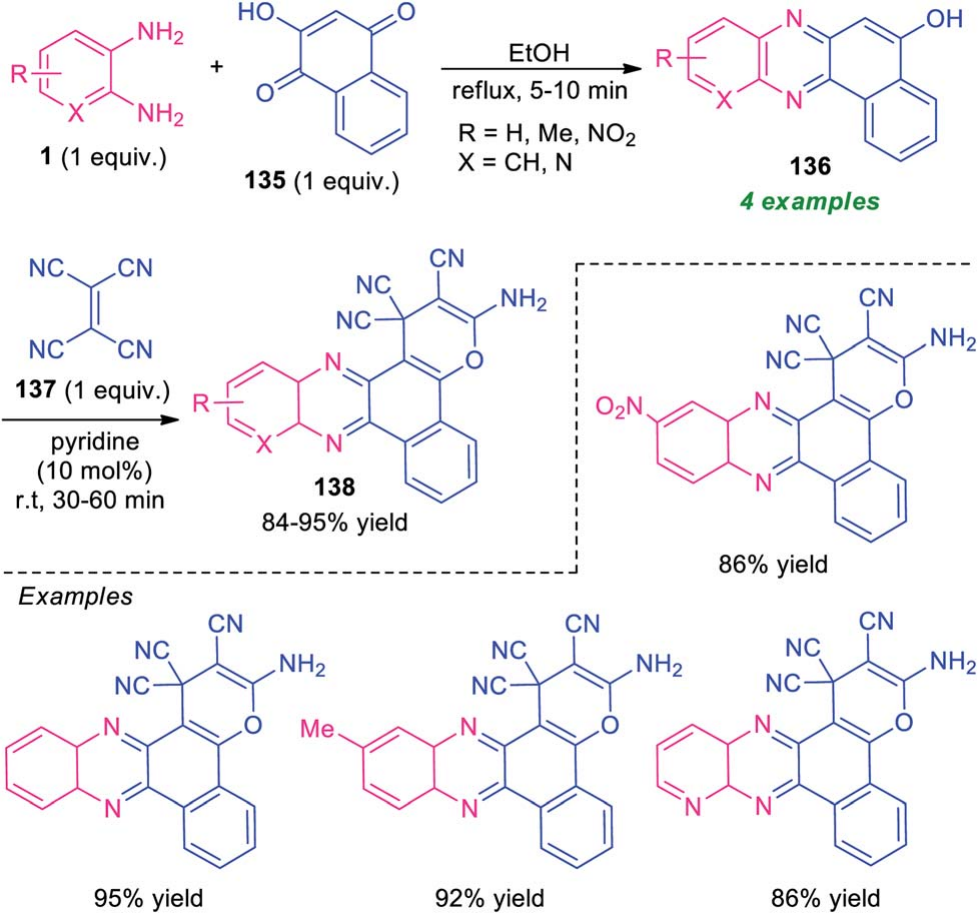
One-pot two-step three-component synthesis of pyran-fused quinoxalines.

A novel one-pot three-component domino reaction between *o*-phenylenediamine 1, 2-hydroxynaphthalene-1,4-dione 135, and tetracyanoethylene 137 using pyridine as a catalyst in ethanol at room temperature was presented by Mohebat and co-authors for the efficient synthesis of pyrano-fused quinoxalines 138 (Scheme 40).^125^ The resulting quinoxalines 138 were obtained in good to high yields. The initial step involving the reaction of 1 with 135 takes place within 5–10 minutes under reflux condition in ethanol to form benzo[*a*]pyrano[2,3-*c*]phenazine 136, which on treating with 137 under the influence of 10 mol% of pyridine afford the final products 138. The products were simply isolated by recrystallization techniques that do not require further conventional purification procedures. Although the presented approach provides several key features like short reaction time, cost-effectiveness, operation simplicity, however, the narrow substrate scopes again constitute a limitation toward this method and calls for further development.

**Scheme 41:**
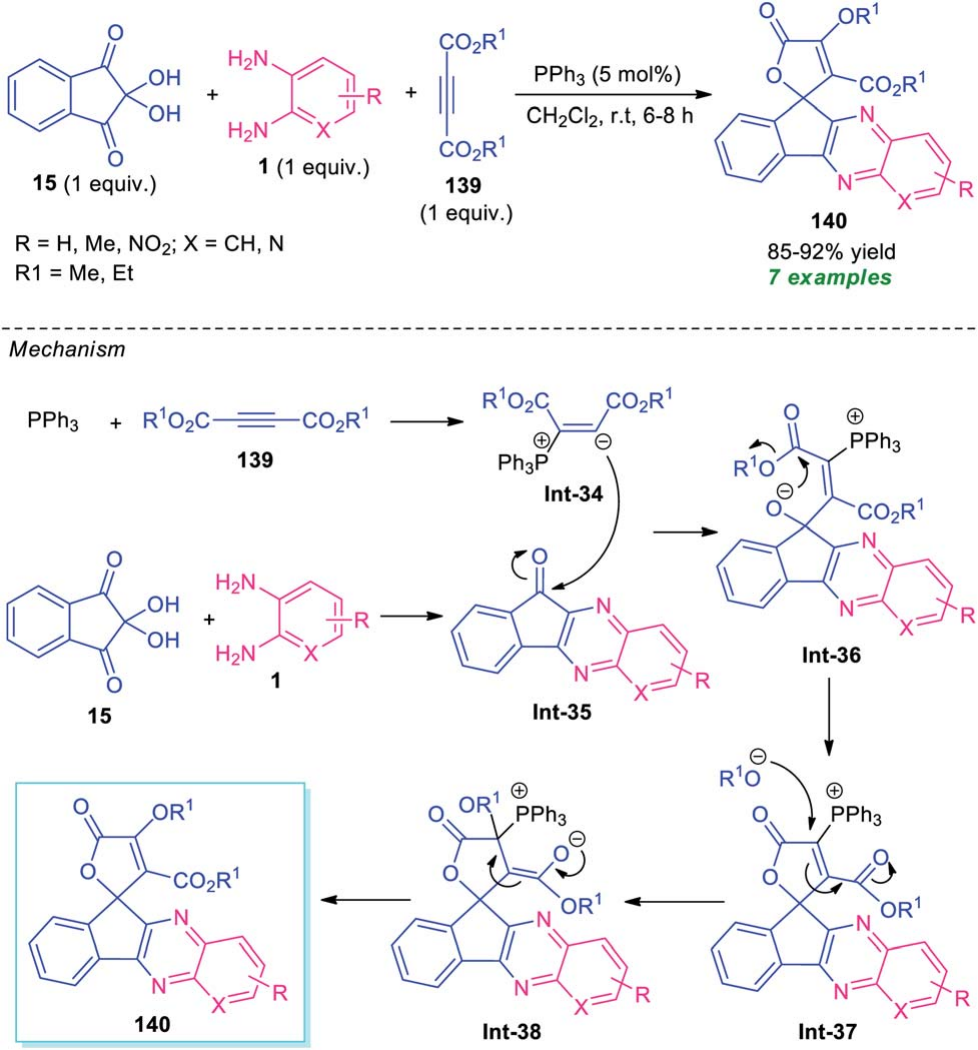
PPh_3_ catalyzed one-pot domino reaction to access spiro-fused quinoxaline 140.

For the construction of diverse annulated quinoxalines, ninhydrin was employed as a versatile and easily accessible starting material. In this regard, Maghsoodlou and co-workers in 2017, disclosed a one-pot multicomponent domino protocol under the metal-free condition to access a variety of spiro-furan-indeno[1,2-*b*]quinoxalines in 85–92% yields by employing ninhydrin 15 as the starting material, alongside *o*-phenylenediamine 1, and dialkyl acetylene dicarboxylate 139 (Scheme 41).^126^ The reaction was performed in presence of 5 mol% of triphenylphosphine (PPh_3_) in CH_2_Cl_2_ at ambient temperature that could be completed within 6–8 hours. In this transformation, PPh_3_ reacts with 139 to produce 1,3-dipolar intermediate Int-34, which eventually attacks the C=O of spiro-indeno[1,2-*b*]quinoxaline Int-35 formed *in situ* from the condensation of 15 and 1. The resulting zwitterionic intermediate Int-36 underwent intramolecular cyclization to form an intermediate Int-37, which is followed by subsequent rearrangement to yield the desired products 140.

**Scheme 42:**
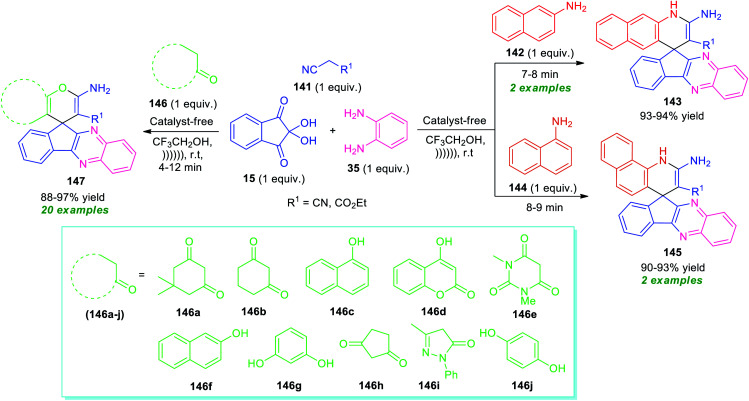
Ultrasound irradiated one-pot construction of several fused quinoxaline derivatives under metal-free condition.

Around the same time, Heravi and Norouzy showed the successful application of ninhydrin in the multicomponent reactions for the synthesis of a library of fused quinoxaline derivatives by employing ultrasonic techniques as a non-conventional strategy (Scheme 42).^127^ With the catalyst-free condition, treatment of ninhydrin 15 with *o*-phenylenediamine 35, active methylene compound 141, and amines 142 or 144 in presence of trifluoroethanol (TFE) under ultrasound irradiation at room temperature provides easy access of spiro[benzo[*g*]quinoline-4,10′-indeno[2,3-*b*]quinoxaline] 143 and spiro[benzo[*h*]quinoline-4,10′-indeno[2,3-*b*]quinoxaline] 145 in high yield respectively. On the other hand, the same reaction condition was found to be very suitable for various CH-activated acidic compounds 146 that react with ninhydrin 15, *o*-phenylenediamine 35, and active methylene compound 141 to form various spiro-fused quinoxalines 147 in good to excellent yield. All the tested CH-activated acidic compounds efficiently worked well under this condition. A comparative study for both conventional and ultrasonic methods revealed that the conventional method utilized a higher amount of energy, as well as a long reaction time as compared to ultrasonic conditions. Conversely, the exploitation of ultrasound not only shortened the reaction time but also improves the yield of the products.

Unlike ultrasonic techniques,^128^ the attractiveness of microwaves^129^ for the synthesis of promising molecular structures has recently gained favour due to its remarkable advantages associated with the synthetic practitioner like mild-condition, reduces reaction times, enhances product yield, and provides high purity of products as well as green chemistry point of view.

**Scheme 43:**
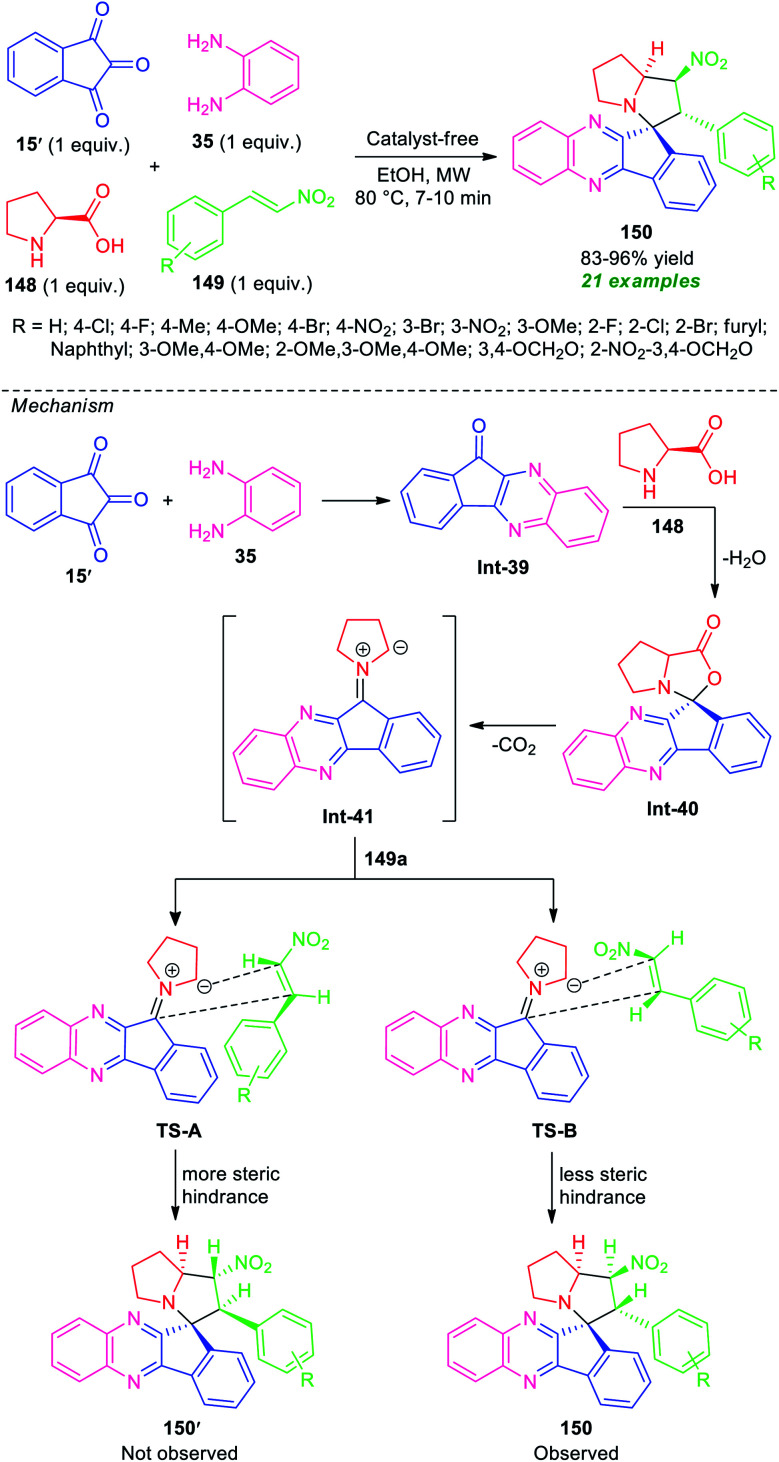
Microwave-assisted regio- and stereoselective one-pot four-component synthesis of spiro-fused quinoxalines.

In 2017, Kantam, Trivedi, and co-authors introduced a microwave technique to develop a convenient strategy for the four-component synthesis of spiro-indeno[1,2-*b*]quinoxaline-11,3′-pyrrolizines derivative 150 based on an azomethine ylide 1,3-dipole mediated [3 + 2]-cycloaddition reaction (Scheme 43).^130^ The synthesis starts with the treatment of ninhydrin 15, *o*-phenylenediamine 35, l-proline 148, and β-nitrostyrene 149 in microwave heating at 80 °C in ethanol, to form the corresponding products 150 as single regio- and diastereomer in good to high yield. Not only aryl-substituted β-nitrostyrene bearing various electron-rich and electron-poor substituents but also heteroaryl as well as alkyl-substituted β-nitrostyrene were well tolerated by this method. The use of conventional heating conditions instead of microwave techniques required a longer reaction time for the completion of the reaction and also a slightly lower yield of the product was observed. The exploitation of microwaves provides a clean reaction profile, shortened the reaction time, and increases the product yield. Other salient features of this approach included broad functionality, eco-friendly, and environmentally benign nature.

To rationalize the stereochemistry of this regioselective dipolar cycloaddition of azomethine ylide Int-41 generated from 15′, 35, and 148 to a dipolarphile 149a, two transition states were proposed, TS-A and TS-B based on steric considerations. As shown in Scheme 43, only the *endo*-product 150 was formed. This is presumably due to the thermodynamically more stable TS-B, while the *exo-*product 150′ was not formed due to more steric repulsion (TS-A).

**Scheme 44:**
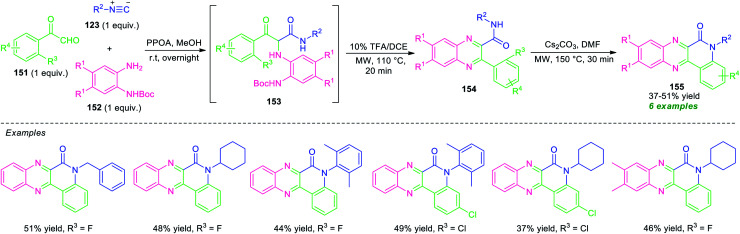
Acid-less Ugi-deprotection-cyclization-substitution sequence for the synthesis of quinolinone-fused quinoxalines.

An acid-free Ugi-deprotection-cyclization (UDC) approach toward the synthesis of quinolinone-fused quinoxalines has been demonstrated by Xu and co-workers (Scheme 44).^131^ The overall process starts with the initial post-Ugi three-component reactions of 2-oxo-2-phenylacetaldehyde 151, *N-Boc*-protected *o*-phenylenediamine 152, and isocyanide 123 under the influence of phenyl phosphonic acid (PPOA) as the catalyst in MeOH at room temperature. The resulting Ugi product 153, on treating with 10 mol% of TFA in 1,2-dichloroethane (DCE) under microwave heating condition at 110 °C, afforded the adduct 154*via* deprotection and cyclization sequence. Subsequently, a nucleophilic substitution reaction of 154 was occurred in presence of Cs_2_CO_3_ in DMF in microwave condition at 150 °C to furnish the final products 155. This multi-component reaction provides a total of six compounds in 37–51% yields from different substituted *o*-phenylenediamine, aryl as well as alkyl-substituted isocyanide and 2-oxo-2-phenylacetaldehyde.

**Scheme 45:**
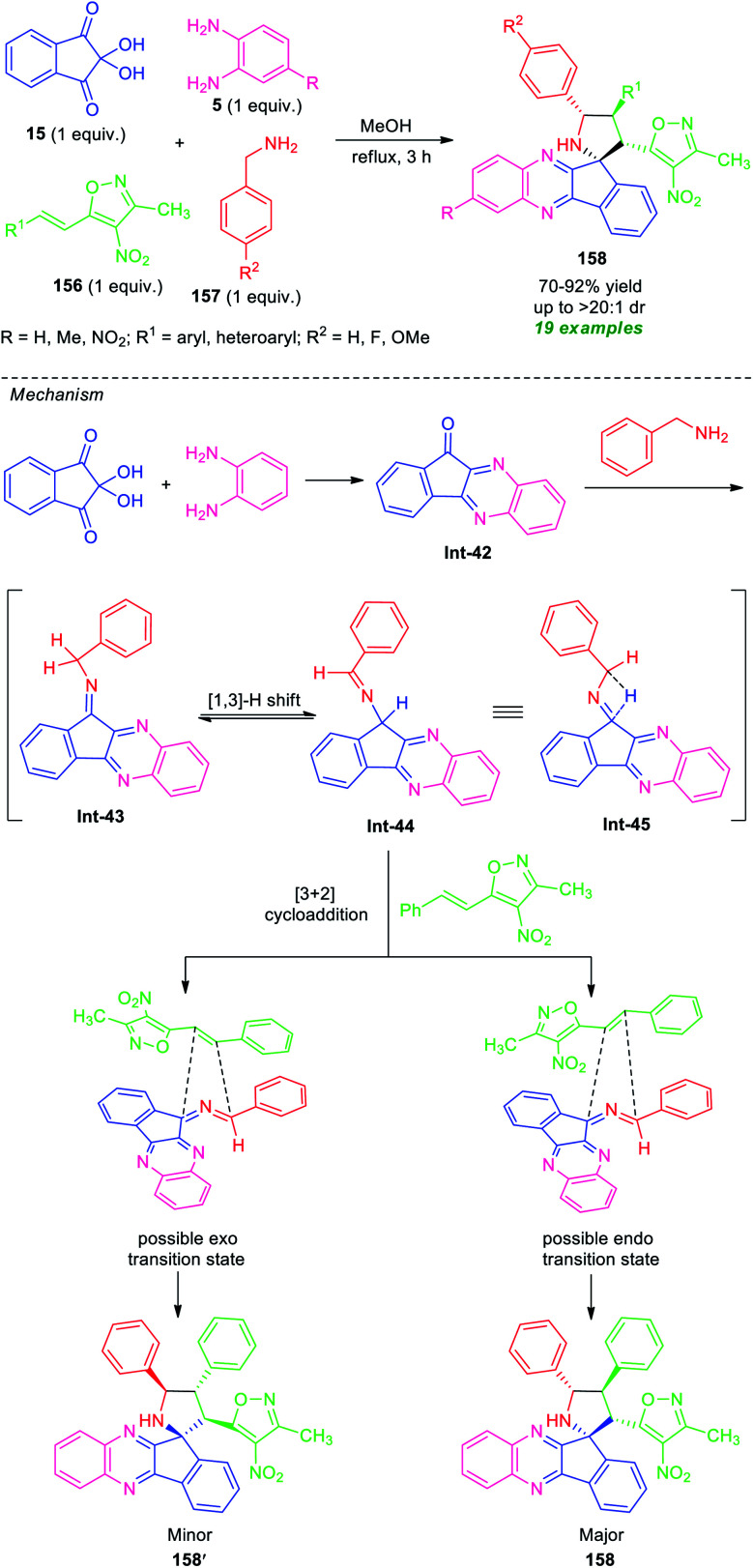
Catalyst-free regio- and diastereoselective one-pot synthesis of spiro-fused quinoxalines.

In 2018, Chowhan *et al.* disclosed a catalyst-free four-component reaction of ninhydrin 15, *o*-phenylenediamine 5, 3-methyl-4-nitro-5-alkenylisoxazoles 156, and benzylamine 157 under reflux condition in methanol for 3 hours to provide a series of spiro-indeno[1,2-*b*]quinoxaline-pyrrolidines 158 (Scheme 45).^132^ This multicomponent approach proceeds *via* an *in situ* generated azomethine ylide 1,3-dipole mediated [3 + 2] cycloaddition reaction. The resultant products were achieved in moderate to high yield with outstanding diastereoselectivity. A variety of differently substituted aryl and heteroaryl rings of 156, as well as unsubstituted or methylated diamines 5, were well tolerated by this method. However, nitro-substituted diamines failed to yield any product. This is presumably due to the electron-poor nature of the nitro group that makes the substrate labile.

Based on a series of observations, a mechanism and transition state to account for the stereochemistry were proposed by the authors. Initial dehydration reaction between ninhydrin, *o*-phenylenediamine, benzylamine provided the isomeric intermediate Int-43 and Int-44 which can be equilibrium to intermediate Int-45. This azomethine ylide type intermediate Int-45, then stereoselectively undergoes [3 + 2] cycloaddition with phenyl-isoxazole *via* two transition states. The *endo* isomer 158 was formed as the major isomer over *exo* product 158′. This can be attributed based on the fact that in 158, the 3-methyl-4-nitroisoxazoles core is positioned closer to the dipolar region that is opposite in 158′.^132^

**Scheme 46:**
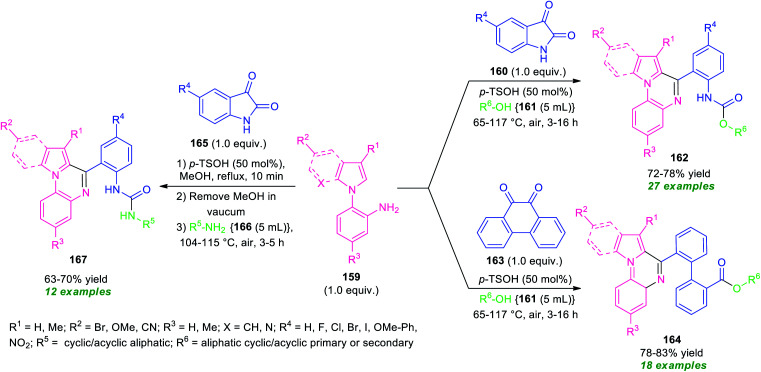
Metal-free solvent-mediated diversity-oriented one-pot domino synthesis of various fused quinoxalines.

Recently, a transition-metal-free one-pot domino process for the synthesis of diverse pyrrolo/indolo[1,2-*a*]quinoxaline derivatives has been realized by Mandal and Pramanik (Scheme 46).^133^ By using 50 mol% of *p*-TsOH as the catalyst, a three-component reaction of *N*-(2-aminophenyl)pyrroles/indoles 159, with various cyclic 1,2-dicarbonyl compounds 160 or 163 and alcohols 161 or aliphatic amines 166 were performed in open-air heating condition. The reactions provide a variety of different fused quinoxaline derivatives 162, 164, and 167 in moderate to good yields. In these reactions, aliphatic amines and alcohols were acted both as solvent as well as the reactant. To explore the generality of the developed protocol, different *N*-(2-aminophenyl)pyrroles/indoles 159 and cyclic 1, 2-dicarbonyl compounds bearing electron-withdrawing as well as electron-donating groups, and amines or aliphatic alcohols were employed. All the substrates were found to be well tolerated. However, tertiary alcohols (*tert*-butyl alcohol and 2,2,2-trifluoroethanol) failed to yield any products by this method. This is presumably due to the steric crowding and reduced nucleophilicity of the free hydroxyl group. The wide substrate scopes, mild reaction condition, metal- and oxidant-free, air as green oxidant are some of the key features of this approach.

## 7. Conclusion and future perspective

Considering the great importance of the quinoxaline framework in many branches of chemistry including natural products chemistry, drug discovery, medicinal chemistry, material science, and agrochemical area, tremendous efforts have been dedicated toward their synthesis. Consequently, many more useful and efficient synthetic protocol has been developed. Synthetic modifications of the classical methods for the preparation of numerous quinoxalines have also emerged. However, most of them are not clearly focused on the development of an efficient route that not only enhances the yield of the product but also reduces the cost of the reaction, provides a clean reaction profile, atom-economic, and utilized an environmentally benign pathway. Therefore, the development of novel synthetic strategies that directs the efficient synthesis of quinoxalines inconsistent with the green and sustainable chemistry principles to make a toxic and waste-free nature are highly desired.

In this pursuit, the last few years have witnessed special attention in the metal-free synthesis of quinoxalines by employing various non-metal inorganic and organic catalysts, ionic liquids, organocatalysts, molecular iodine, catalyst-free condition, green catalyst, visible-light-induced and non-conventional strategies like microwaves, and ultrasound irradiation. The exploitation of these reagents and techniques certainly makes these protocols environmentally as well as eco-friendly benign compared to those transition-metal-catalyzed routes. Despite, notable developments achieved in the transition-metal catalyzed synthesis, high cost involved in the preparation of the catalyst, toxicity, and difficulty in removing it from the final products constitutes disadvantageous effects on the atom economy and eco-friendly nature of the transformation.

In this review article, we have summarized the recent progress achieved in the synthesis of quinoxalines and various fused quinoxalines by employing two-component and multicomponent reactions under metal-free conditions and cover the reports from 2015 to date. This aspect is presented alongside the mechanistic rationalization and limitation of the reaction methodologies. The scopes of future developments are also highlighted.

As illustrated through the review, it is clear that these protocols have significant advantages such as a high level of yield, broad functional group tolerance, mild reaction condition, cost-effectiveness, *etc.* Other key features included the utilization of water as green solvents over other toxic and volatile solvents due to its remarkable properties like wide abundance, non-toxic, and inexpensive nature. Besides these, solvent-less protocols are also developed under metal-free conditions. In addition to these, the utilization of microwaves and ultrasonic techniques shortened the reaction times as well as enhanced the reactivity and selectivity of the reaction, and provides an excellent yield of the quinoxaline products.

On the other hand, the combination of the metal-free condition along with multicomponent reactions opened a new gateway for the easy access of highly functionalized quinoxalines.

Despite, notable advancement achieved in the metal-free synthesis of quinoxalines, limited substrate scopes, requirements of higher energy source, low yield of the products, longer reaction time, and high catalyst loading, constitutes the major limitations of some so far developed protocols. Therefore, ample attention needs to be paid in the upcoming days for broadening the substrate scopes with excellent yields by employing mild pathways which are safe, eco-benign, atom-economic, and provide scale-up synthesis in low catalyst loading or catalyst and metal-free condition for application to the industrial area. Also, the biological as well as material science applications of novel quinoxaline derivatives need to be screened and should be strengthened. We hope, the present review article will help researchers working on this fascinating area for the design and developments of more precise synthetic routes for the construction of quinoxalines and for further outstanding creation of a highly functionalized novel quinoxaline framework that would find immense application in the field of medicinal chemistry and optoelectronic materials.

## Conflicts of interest

“There are no conflicts to declare”.

## Supplementary Material
